# An Example-Based Multi-Atlas Approach to Automatic Labeling of White Matter Tracts

**DOI:** 10.1371/journal.pone.0133337

**Published:** 2015-07-30

**Authors:** Sang Wook Yoo, Pamela Guevara, Yong Jeong, Kwangsun Yoo, Joseph S. Shin, Jean-Francois Mangin, Joon-Kyung Seong

**Affiliations:** 1 Department of Biomedical Engineering, Korea University, Seoul, Republic of Korea; 2 Department of Computer Science, KAIST, Daejeon, Republic of Korea; 3 I^2^BM, CEA, Gif-sur-Yvette, France; 4 Institut Fédératif de Recherche 49, Gif-sur-Yvette, France; 5 University of Concepción, Concepción, Chile; 6 Department of Bio and Brain Engineering, KAIST, Daejeon, Republic of Korea; 7 Handong Global University, Pohang, Republic of Korea; Istituto Italiano di Tecnologia, ITALY

## Abstract

We present an example-based multi-atlas approach for classifying white matter (WM) tracts into anatomic bundles. Our approach exploits expert-provided example data to automatically classify the WM tracts of a subject. Multiple atlases are constructed to model the example data from multiple subjects in order to reflect the individual variability of bundle shapes and trajectories over subjects. For each example subject, an atlas is maintained to allow the example data of a subject to be added or deleted flexibly. A voting scheme is proposed to facilitate the multi-atlas exploitation of example data. For conceptual simplicity, we adopt the same metrics in both example data construction and WM tract labeling. Due to the huge number of WM tracts in a subject, it is time-consuming to label each WM tract individually. Thus, the WM tracts are grouped according to their shape similarity, and WM tracts within each group are labeled simultaneously. To further enhance the computational efficiency, we implemented our approach on the graphics processing unit (GPU). Through nested cross-validation we demonstrated that our approach yielded high classification performance. The average sensitivities for bundles in the left and right hemispheres were 89.5% and 91.0%, respectively, and their average false discovery rates were 14.9% and 14.2%, respectively.

## Introduction

Diffusion of water molecules in cerebral white matter (WM) is anisotropic, reflecting its organization in bundles of WM tracts running in parallel. 3D trajectories of WM tracts can be reconstructed from diffusion-weighted image data using tractography algorithms [1–5]. The reconstructed trajectories for known deep WM bundles agree well with results in postmortem anatomic studies [6]. The resulting WM tracts can be classified into a set of well-known anatomic bundles, which have been used in several studies on pathologies [7–12] and neurodevelopment [13, 14].

Since neural tracts are conducting electrical impulses, damage to neural tracts will lead to disrupt the impulse propagation. Neural tract damage induced by certain pathologies might affect diffusion properties. Diffusion-based MRI metrics such as fractional anisotropy (FA) and mean diffusivity (MD) have been widely used to evaluate WM microstructural integrity in health and disease. For example, in patients with multiple sclerosis, a drop of the FA value was observed along the corpus callosum, which is one of the major anatomic bundles [8]. Also, diffusion properties change along with the development: observing diffusion properties of major bundles of 1 and 2 year old subjects, Goodlett et al. showed that the FA value of 2 year old subjects is greater than that of 1 year old subjects [14]. The classification of input WM tracts into anatomic bundles facilitates the effective comparison of the same anatomic bundles across different subjects, in tract-based studies [15, 16].

Well-known anatomic bundles have been obtained by delineating in MR images some regions of interest (ROIs), through which the bundles pass [1, 3, 17–19]. These methods share a limitation of manual specification of ROIs, which is cumbersome and time-consuming. Moreover, ROI specification requires expert knowledge on anatomy of WM bundles. Recently, several automatic classification techniques have been introduced, based on brain registration to an atlas [16, 20–22]. Zhang et al. [21] automatically labeled ROIs by employing the large deformation diffeomorphic metric mapping (LDDMM) for non-linearly warping an atlas with ROIs to an individual subject data. However, the variability of neural tract patterns over individual subjects has not been taken into account, since these methods use a single atlas-based registration scheme. We aim to obtain more accurate classification results by exploiting the individual variability of subjects.

There have been approaches to clustering the WM tracts based on their shape [23–29]. In O’Donnell et al. [23], spectral clustering was employed to classify the input tracts into bundles according to their shape similarity. For details of spectral clustering, we refer readers to [30]. Guevara et al. [24, 25] identified major bundles which commonly exist in most of subjects using a two-step method, consisting of intra-subject clustering followed by inter-subject clustering. The inter-subject clustering method [25] refined bundles by first classifying the tracts into fascicles using tract extremities and then merging these fascicles based on the pairwise distances between their centroids. The above methods have the common limitation that resulting bundles (clusters) require the manual labeling by experts.

Several works employed an atlas with expert-provided knowledge for automatic labeling of WM tracts [22, 31–36]. Bazin et al. [22] used an atlas called a WM tract atlas to automatically label WM tracts. In this atlas, every voxel contains the information on the shapes and directions of tracts passing through it. The probability of a voxel label was estimated based on the Markov random field modeling of diffusion. The label of an input tract is determined according to the probability of voxel labels that the tract passes through. A single atlas was used for registration of an input DTI of a subject, which may not reflect the individual variability of bundles over subjects. To take into account the variability, O’Donnell and Westin [31] constructed an atlas with WM tracts from multiple subjects. Specifically, the tracts of example subjects were grouped based on spectral clustering, and the resulting clusters were labeled by experts to create an anatomic atlas of WM bundles. Then, the tracts of a test subject were automatically classified by mapping them onto the atlas space and finding the closest cluster center to inherit its anatomic label. However, this method is quite computationally demanding even with a sampling scheme called the Nystrom method [37]. We avoided spectral clustering to achieve great computational efficiency by compactly representing WM bundles while employing multiple atlases to label input tracts, which is facilitated by simultaneous labeling of similar input tracts. Furthermore, our approach shows better accuracy than a ROI-based automatic labeling method [21]. Recently, Jin et al. employed multiple atlases, each of which consists of hand-labeled major tracts for a subject [35]. The manual atlases were warped to the test subject’s space, and an ROI-based clustering and a distance-based clustering are sequentially applied to label the tracts of the test subject. However, the ROI-based clustering could remove some tracts of interested anatomic bundles due to the inaccuracy in the registration stage, as also shown in our experiments (see the Results and Discussion section). Tunc et al. also proposed a multiple atlases-based approach for labeling WM tracts [36]. Their approach used a fiber representation based on the connectivity between parcellated brain regions, whose accuracy may depend on the registration scheme. Also, the proposed representation may not reflect the tract shapes exactly because it is based on the connectivity information. Thus, spurious tracts could be included in the clustering results.

Guevara et al. [33] provided an example-based single-atlas approach for labeling the tracts of a subject, based on expert-provided example data from multiple subjects. Their approach combined the multi-subject example data into a single atlas in advance. Recently, Labra et al. proposed a fast version of the Guevara et al.’s approach [38]. We compared the labeling accuracies of our method with the Guevara et al.’s method, and our method showed better accuracies than their method. We present here an example-based multi-atlas approach, which is simple, flexible, and efficient. Our approach is conceptually simple by employing the same metrics in both example data clustering and tract labeling, while avoiding complicated voxel parcellation and inter-subject clustering. It is also flexible in adding and deleting the example data of a subject, by maintaining an atlas for each subject. We employ a voting scheme for labeling the tracts of a given subject to facilitate the multiple atlas consultation. This voting scheme also facilitates removing the outliers in the labeling results.

The contributions of this paper are three-fold: First, we construct multiple atlases from different subjects in order to consider the individual variability of bundle shapes and trajectories. An atlas is maintained for each example subject to allow the example data of a subject to be added or deleted flexibly. Unlike similar existing methods [35, 36], our method does not rely on the ROI-based scheme which can cause inaccuracies due to the registration error. A voting scheme is proposed to facilitate the multi-atlas exploitation of example data and to effectively remove outliers from the labeling results. Second, for conceptual simplicity, we adopt the same metrics in both example data construction and WM tract labeling. This eventually leads to a labeling scheme, which is simple and easy to implement. Finally, we greatly enhance computational efficiency for labeling WM tracts. Due to the huge number of tracts in a subject, it is time-consuming to label each tract individually. Thus, the tracts are grouped according to their shape similarity, and tracts within each group are labeled simultaneously. To further enhance the computational efficiency, we implemented our approach on the GPU.

## Materials and Methods

### MRI acquisition and preprocessing

We validate our approach with the brain image data from twelve subjects in the NMR public database [39]. The data is the property of CEA Neurospin and can be provided on demand to cyril.poupon@cea.fr. All subjects are male and their ages are ranged from 21 to 40 years (mean±standard deviation: 32.8±5.5). This database provides high quality T1-weighted images and diffusion data acquired with a GE Healthcare Signa 1.5 Tesla Excite II scanner. The diffusion data presents a high angular resolution (HARDI), based on 200 directions and a b-value of 3000 s/mm^2^ (voxel size of 1.875 mm × 1.875 mm × 2 mm). Diffusion-weighted data were acquired using a twice refocusing spin echo technique [40] compensating Eddy currents to the first order. Geometrical distortions due to susceptibility artifacts were corrected using a phase map acquisition. T1 and diffusion-weighted data were optimally aligned using a rigid 3D transform estimated by an automatic registration algorithm based on mutual information. Registration was performed between the average of five diffusion-free T2-weighted images and the high resolution T1-weighted image.

### WM tract extraction

Given a set of diffusion-weighted images (voxel size of 1.875 mm × 1.875 mm × 2 mm) for an example subject, the diffusion Orientation Distribution Function (dODF) is estimated from an analytical solution for Q-ball imaging with a spherical harmonic basis (spherical harmonic order 4 and Laplace-Beltrami regularization *λ* = 0.006), as described in [41]. The dODF gives a probability density function at every voxel which characterizes water diffusion along any given direction [42]. We employ a deterministic tractography algorithm [43] in the BrainVISA software (http://brainvisa.info) to reconstruct WM tracts from the dODF sampled at every voxel center. To prevent the tracts from penetrating cortical folds, we use a mask of WM which is built based on a T1-weighted image [44]. Two seeds are localized in each voxel of the WM mask (voxel size of 0.9375 mm × 0.9375 mm × 1.2 mm) in order to initiate WM tract tracking. A single tract is tracked at a seed in both retrograde and orthograde directions with respect to the maximal direction of the dODF data, and the results are combined to obtain a tract. The tracking is stopped when the tract exits the WM mask, when the angle between the two last moves exceeds 30°, or when the tract length exceeds 200 mm. About 1.5 million of tracts per example subject are stored after filtering out the tracts shorter than 20 mm.

Brains of different subjects may be of different sizes and have different positions and orientations when they are scanned. In order to globally align the diffusion-weighted images and, accordingly, the WM tracts from different subjects, a single example subject is selected as a reference subject, and an affine transformation from each example subject to the reference subject is computed through image registration between their T1-weighted images. We used the affine transformation for subject registration because transforming WM tracts using highly-nonlinear transformation may produce unexpected distortion of tract shape. The affine transformations between the example subjects and the reference subject were estimated by using the Linear Image Registration Tool (FLIRT) of the FMRIB Software Library (FSL) [45]. The resulting registration matrix was used for transforming the WM tracts of each example subject into the space of the reference subject.

### Overview of the method

**Fig 1:**
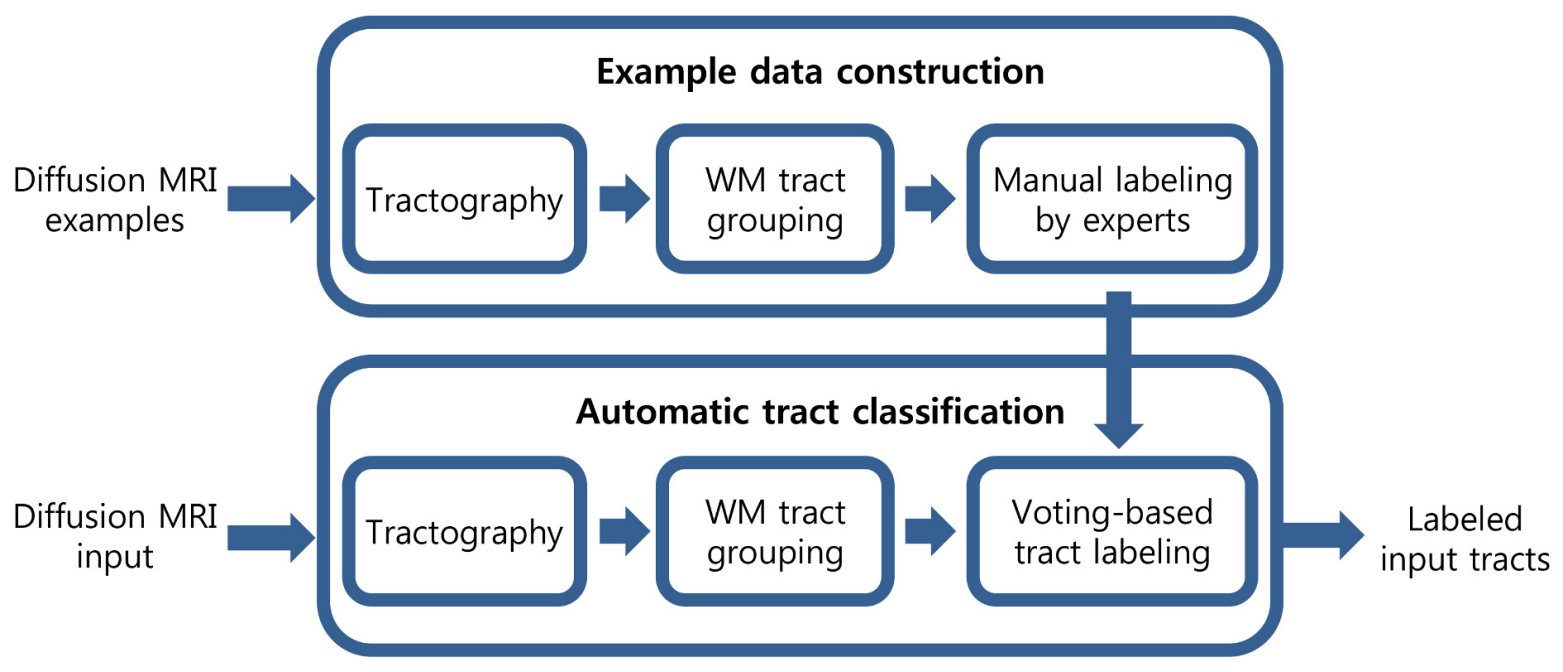
Overview of the proposed approach to automatic classification. The method consists of two parts: Example data construction and automatic tract classification.

In the following sections, we present an example-based multi-atlas approach for automatic classification of WM tracts using example anatomic bundles which are labeled by experts. As shown in Fig 1, our approach for automatic classification consists of two steps: example data construction and automatic tract classification. In the former step, input tracts, which are obtained from the tractography algorithm, are manually classified by experts into seven anatomic bundles for each hemisphere of example subjects. Since the number of tracts is too huge, they are first grouped according to their shape similarity to help the manual labeling. In the latter step, the input tracts are automatically classified into anatomic bundles by exploiting the example bundle data. For an efficient classification, the input tracts of a subject are also clustered as in the former step. For each input group, an example bundle containing the nearest tract group is chosen in terms of Kullback-Leibler divergence (KLD) in order to inherit its label to all tracts in the group. We propose a voting-based approach for more robust classification. To handle a large number of tracts efficiently, we implemented our approach using GPU.

### Example data construction

#### WM bundle modeling

The 3D curves resulting from tractography are usually referred to as fibers, although they do not represent individual nerve fibers. Actually, these curves estimate 3D trajectories of fiber pathways. In this paper, the trajectories which are obtained with tractography will be referred to as “tracts”. Thus, a “WM bundle” is a bundle of curves that are obtained from tractography but not a bundle of actual nerve fibers. Labeling of tracts is giving anatomic names to them. As shown in Table 1, we classify input tracts into seven anatomic bundles which have been commonly used in previous works [16, 20–22, 31].

**Table 1:** Major anatomic bundle list.

Symbols	Labels	Remarks
ATR	Anterior thalamic radiation	Connection between the thalamus and the prefrontal cortex
CST	Corticospinal tract	Connection between the cerebral cortex and the spinal cord
CG	Cingulum	Projection from the cingulate gyrus to the entorhinal cortex
IFO	Inferior fronto-occipital fasciculus	Connection between the frontal and the occipital lobes
ILF	Inferior longitudinal fasciculus	Connection between the occipital and the temporal lobes
SLF	Superior longitudinal fasciculus	Connection from the frontal lobe to the occipital, and part of the parietal and the temporal lobes
UNC	Uncinate fasciculus	Connection from the anterior temporal lobe to the orbital cortex

A tract is a piecewise linear curve represented as a sequence of points. We obtained the 3D B-spline curve passing through these points to reparameterize the curve based on its chord length. By uniform sampling along the curve, every tract is then represented as a sequence of sample points. Therefore, a single tract with *n* sample points can be represented as a point *f* in the 3*n*-dimensional space as follows:

$$\begin{array}{c} f={\left(x_{1},y_{1},z_{1},x_{2},y_{2},z_{2},\cdots ,x_{n},y_{n},z_{n}\right)}^{T} \end{array}$$ (1)

where (*x*_*i*_, *y*_*i*_, *z*_*i*_) is the three-dimensional Cartesian coordinates of the *i*-th sample point. This fixed-vector representation of a tract has been used to simplify the point-wise tract correspondence problem [46]. The similarity between two tracts is simply measured by Euclidean distance between their equivalents in the 3*n*-dimensional space.

$$\begin{array}{c} d_{E}\left(f_{i},f_{j}\right)=\sqrt{{\left(f_{i}-f_{j}\right)}^{T}\left(f_{i}-f_{j}\right)} \end{array}$$ (2)

where *f*_*i*_ and *f*_*j*_ are two tracts which are represented as 3*n*-dimensional points, and *d*_*E*_ denotes the Euclidean distance function.

**Fig 2:**
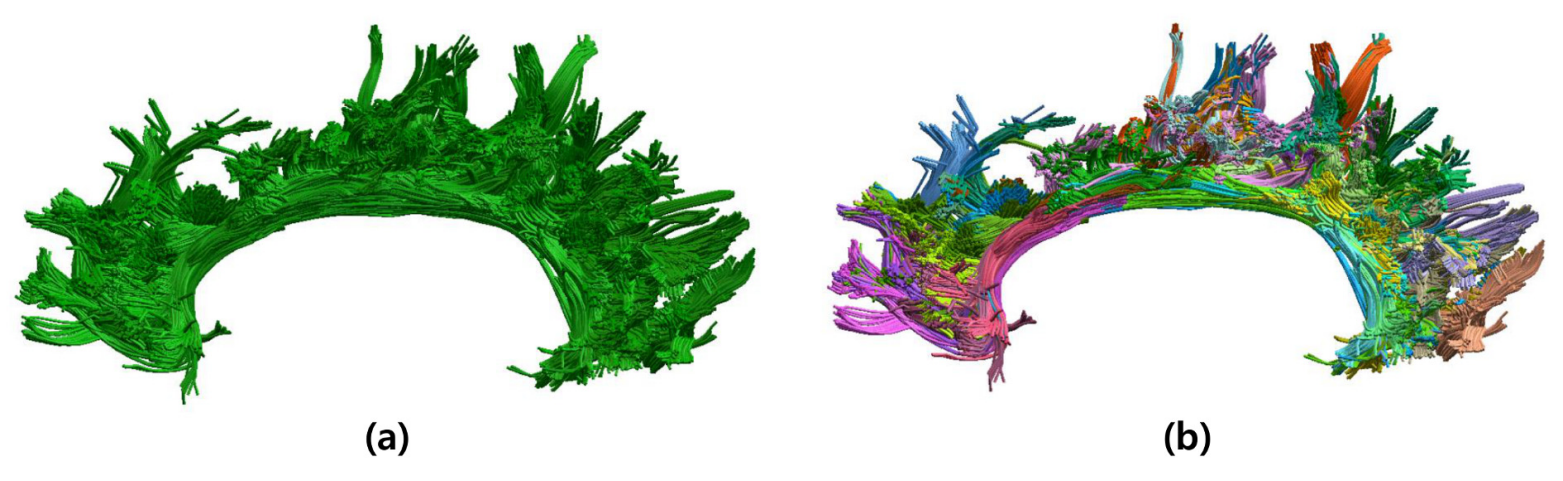
An anatomic bundle consists of tracts with various shapes. Cingulum bundle (a) contains many tracts with different shapes as shown in (b).

As shown in Fig 2, a bundle consists of tracts with various shapes. For convenience, tracts within a bundle are classified into groups according to their shape similarity, and thus a bundle is composed of tract groups. For representation simplicity and also labeling efficacy, each group is modeled with a multivariate Gaussian distribution. A mean vector of tracts within a group is calculated as follows:

$$\begin{array}{c} m=\frac{1}{N}\sum_{i=1}^{N}f_{i} \end{array}$$ (3)

where *N* is the number of tracts in the group. The covariance matrix can be estimated with a well-known empirical unbiased covariance matrix.

$$\begin{array}{c} S=\left[s_{ij}\right]=\frac{1}{N-1}\sum_{k=1}^{N}\left(f_{k}-m\right){\left(f_{k}-m\right)}^{T} \end{array}$$ (4)

However, the covariance matrix *S* becomes singular or near-singular if the number of tracts in the group is less than 3*n* or many tracts are nearly parallel to each other. To overcome this problem, we adopt a shrinkage estimation method [47] to find a substitute matrix S* as follows:

$$\begin{array}{c} S^{*}=\left[s_{ij}^{*}\right]=\{\begin{array}{cc} s_{ii} & \text{if}\;i=j\\ r_{ij}^{*}\sqrt{s_{ii}s_{jj}} & \text{if}\;i\neq j \end{array} \end{array}$$ (5)

and

$$\begin{array}{c} r_{ij}^{*}=\{\begin{array}{cc} 1 & \text{if}\;i=j\\ r_{ij}min\left(1,max\left(0,1-\hat{\lambda^{*}}\right)\right) & \text{if}\;i\neq j \end{array} \end{array}$$

with

$$\begin{array}{c} \hat{\lambda^{*}}=\frac{\sum_{i\neq j}^{}\hat{Var}\left(r_{ij}\right)}{\sum_{i\neq j}^{}r_{ij}^{2}} \end{array}$$

where *s*_*ii*_ and *r*_*ij*_ are empirical unbiased variance and correlation respectively, and $\hat{Var}(r_{ij})$ is an empirical unbiased variance of *r*_*ij*_. The new matrix S* is guaranteed to have a full rank regardless of sample data. The similarity between a tract *f*_*i*_ and a group *B*_*j*_ that is modeled with the multivariate Gaussian distribution can be calculated using the Mahalanobis distance *d*_*M*_:

$$\begin{array}{c} d_{M}\left(f_{i},B_{j}\right)=\sqrt{{\left(f_{i}-m_{j}\right)}^{T}{\left(S_{j}^{*}\right)}^{-1}\left(f_{i}-m_{j}\right)} \end{array}$$ (6)

where *m*_*j*_ and $S_{j}^{*}$ are mean vector and covariance matrix of *B*_*j*_ respectively.

#### WM tract grouping

Since there are a huge number of tracts in each subject, it is tedious and time-consuming to label those tracts individually. Thus, the tracts are grouped according to their shape similarity, and those in the same group are labeled simultaneously.

In order to cluster the tracts of each subject according to their shape similarity, we mainly rely on hierarchical clustering [48] since we could determine the number of clusters by choosing the actual distance (in *mm*) between two neighboring clusters. Because we use huge tract datasets for each subject, the tracts are first clustered into a smaller number of different length ranges, by employing k-means clustering [49], and then hierarchical clustering is employed to group tracts for each length range. Directly applying the hierarchical clustering is practically difficult because the method needs to construct a pairwise distance table, which is too big to fit in a conventional memory. Finally, the similar tract groups in each pair of consecutive length ranges are merged together for completing WM tract grouping. Recently, Garyfallidis et al. proposed a memory efficient and very fast method for clustering a huge number of input tracts [50]. However, the clustering results of the proposed method depend on the initial permutation of input tracts because it is a greedy algorithm. Our method can give consistent clustering results with high accuracies compared to the conventional hierarchical clustering algorithm regardless of initial permutation of input tracts. Furthermore, the memory issue can be handled with the proposed combined clustering approach.

To apply the hierarchical clustering, we first need to determine how to compute distances between two tracts and those between two groups. Since the sample point sequences for two tracts *f*_*i*_ and *f*_*j*_ may not be aligned, the distance between two tracts is calculated as follows:

$$\begin{array}{c} d\left(f_{i},f_{j}\right)=min\left(d_{E}\left(f_{i},f_{j}\right),d_{E}\left(f_{i},f_{j}^{\prime }\right)\right) \end{array}$$ (7)

where $f_{j}^{\prime }$ is a 3*n*-dimensional point obtained from *f*_*j*_ by reversing its sample point sequence. For calculating the distance between two groups *c*_*i*_ and *c*_*j*_, we adopt the average linkage criterion *d*(*c*_*i*_, *c*_*j*_) defined as follows:

$$\begin{array}{c} d\left(c_{i},c_{j}\right)=\frac{1}{|c_{i}\left|\right|c_{j}|}\sum_{f_{i}\in c_{i}}\sum_{f_{j}\in c_{j}}d\left(f_{i},f_{j}\right) \end{array}$$ (8)

where ∣*c*_*i*_∣ is the number of tracts within the group *c*_*i*_, and *f*_*i*_ is a tract that belongs to the group *c*_*i*_. The hierarchical clustering with average linkage has the advantage of robustness to outliers [51].

In order to merge the similar tract groups in a pair of consecutive length ranges, the mean curve of each group is computed as its representative curve. For this purpose, tracts within each group are first aligned so the sequence of sample points are stored following the same orientation: A random tract in the group is first chosen as the reference tract. For each remaining tract *f*, its sample point sequence is then aligned with respect to the reference tract *f*_*r*_ by comparing two distances *d*_*E*_(*f*, *f*_*r*_) and *d*_*E*_(*f*′, *f*_*r*_):

$$\begin{array}{c} \hat{f}=\{\begin{array}{cc} f & \text{if}\;d_{E}\left(f,f_{r}\right)\leq d_{E}\left(f^{\prime },f_{r}\right)\\ f^{\prime } & otherwise \end{array} \end{array}$$ (9)

With the new set of aligned tracts $\hat{f}$, the mean curve of the group can be computed using Eq (3). The mean curves are grouped according to their shape similarity using the average-linkage hierarchical clustering. Finally, the tracts corresponding to the same mean curve group are merged to form a new tract group.

Afterwards, outliers are identified as those groups whose number of tracts is smaller than a threshold value [52]. We try to reassign every tract in each outlier group to a non-outlier group. If the distance between a tract in a outlier group and its closest non-outlier group is greater than a pre-defined distance threshold, the tract is considered as an outlier and removed. Otherwise, it is reclassified to its closest group. The distance between a tract *f*_*i*_ and a tract group *g*_*j*_ is measured as follows:

$$\begin{array}{c} d\left(f_{i},g_{j}\right)=min\left(d_{M}\left(f_{i},g_{j}\right),d_{M}\left(f_{i}^{\prime },g_{j}\right)\right) \end{array}$$ (10)

where *d*_*M*_ is the Mahalanobis distance that is defined in Eq (6). For a *k*-dimensional multivariate Gaussian distribution, it is known that the squared Mahalanobis distance of a data to the mean of the distribution follows the Chi-square distribution with *k* degrees of freedom. In the Chi-square distribution, a critical value for a given confidence level is chosen as the threshold value to filter out a outlier tract.

#### Manual labeling of WM tracts

**Fig 3:**
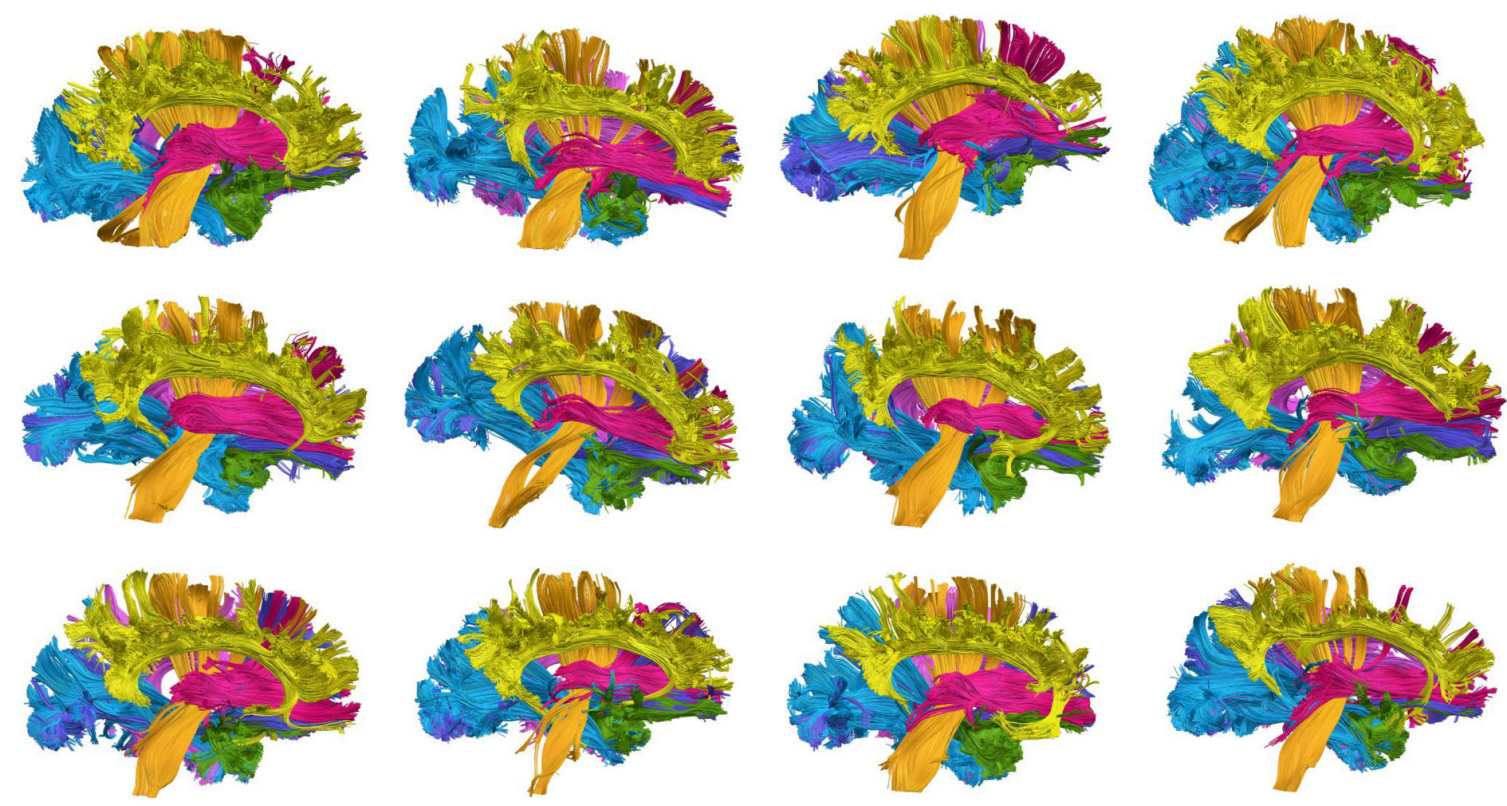
Seven anatomic bundles of twelve example subjects. Seven anatomic bundles which were obtained through manual labeling for left hemispheres of twelve example subjects.

In order to construct the expert-provided example data, the tract groups of each subject are manually labeled by experts based on neuroanatomic knowledge. Specifically, two experts independently classified the tract groups of example subjects into the seven anatomic bundles listed in Table 1, by using an interactive tract labeling tool, designed especially for this task. The label of a group was defined if the two experts gave an identical label. Otherwise, they together reexamined this group for arriving to an agreement. Fig 3 shows seven anatomic bundles which were manually labeled by the experts for the left hemispheres of twelve example subjects. We focus on the seven anatomic bundles since other bundles were not consistent over example subjects due to individual variability.

### Automatic tract classification

#### Basic idea

Tract groups of a subject, which are obtained as explained in *WM tract grouping* section, are used as input for classification. Before classification, tracts of the test subject are transformed to the coordinate frame of the reference example subject through image registration between T1-weighted images. Then, each tract group is labeled by searching the most similar tract group in the example data.

To measure the similarity between an input tract group and each group in the example data, we use a measure based on the KLD [53], which is commonly used for measuring the similarity between two probability distributions. The KLD between two distributions *b*_*i*_ and *b*_*j*_ is defined as follows:

$$\begin{array}{c} d_{KL}\left(b_{i},b_{j}\right)=\int_{-\infty }^{\infty }p\left(x\right)ln\frac{p(x)}{q(x)}dx \end{array}$$ (11)

where *p*(*x*) and *q*(*x*) are probability density functions of *b*_*i*_ and *b*_*j*_, respectively. Since the KLD is non-symmetric, we use the symmetric KLD (SKLD) as the similarity measure, which is defined as follows:

$$\begin{array}{c} d_{SKL}\left(b_{i},b_{j}\right)=d_{KL}\left(b_{i},b_{j}\right)+d_{KL}\left(b_{j},b_{i}\right) \end{array}$$ (12)

It is known that the KLD between two multivariate Gaussian distributions *b*_*i*_ and *b*_*j*_ is computed as follows [54]:

$$\begin{array}{c} d_{KL}\left(b_{i},b_{j}\right)=\frac{1}{2}\left(trace\left(S_{j}^{-1}S_{i}\right)+v^{T}S_{j}^{-1}v-ln\left(det\left(S_{j}^{-1}S_{i}\right)\right)-k\right) \end{array}$$ (13)

where *v* = *m*_*j*_−*m*_*i*_, *m*_*i*_ and *S*_*i*_ are the mean vector and covariance matrix of *b*_*i*_, respectively, and *k* is the dimensions of a point representing a tract. From Eqs (12) and (13), the SKLD between the two multivariate Gaussian distributions is computed as follows:

$$\begin{array}{c} d_{SKL}\left(b_{i},b_{j}\right)=\frac{1}{2}\left(trace\left(S_{i}^{-1}S_{j}\right)+trace\left(S_{j}^{-1}S_{i}\right)+v^{T}S_{i}^{-1}v+v^{T}S_{j}^{-1}v-2k\right) \end{array}$$ (14)

#### Voting scheme

For robustness, we propose a voting scheme with multiple example subjects: given an input tract group, we first determine the most similar group in each example subject, and then every example subject votes for the bundle containing the most similar example group in it. The label of the bundle with the majority of votes is chosen as the label of the input group and inherited to every tract constituting the input group, as long as the number of votes is greater than a given threshold.

**Fig 4:**
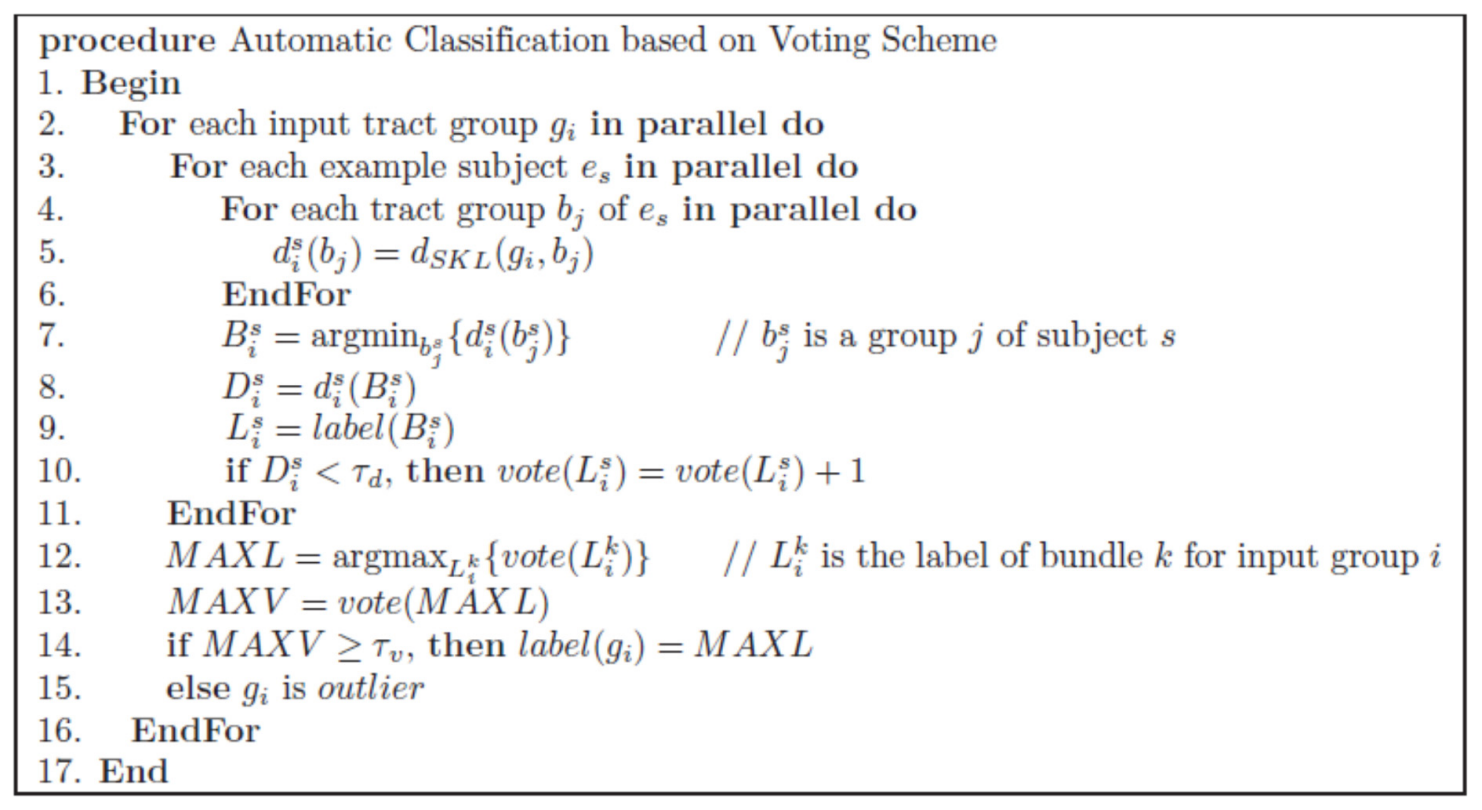
Skeleton of our automatic classification algorithm. The skeleton of the voting algorithm to determine the label of an input group.

Fig 4 shows a skeleton of the voting algorithm to determine the label of an input group. We implemented our algorithm using GPU (See S1 Text for detailed explanation). If the SKLD between an input tract group and its closest group in an example subject is larger than a threshold distance *τ*_*d*_, the subject is not qualified to vote since there is no similar example group to the input group in the example subject. In addition, if the maximum number of votes among all bundles is smaller than a threshold vote number *τ*_*v*_, then the input group is regarded as an outlier.

#### Direct tract classification

Apart from the proposed method for labeling input tract groups, we also propose a method for labeling individual tracts directly. The sequential tract labeling without clustering would take much computation time. However, the computation time can be greatly reduced if we label multiple tracts simultaneously using GPU. We can also remove the computation time of tract grouping by performing the direct tract classification.

For the direct tract classification, we only need to change the distance between two tract groups (Eq (12)) to the distance between a tract *f*_*i*_ and a tract group *b*_*j*_, which can be computed using the Eq (6).

### Performance analysis

In this paper, we use four different measures for assessing the performance of the proposed method: consistency, sensitivity, false discovery rate, and kappa value. We explain each of them in detail below.

#### Consistency

In *WM tract grouping* section, we proposed a new method for grouping a massive number of tracts. In order to show that our grouping method is not dependent on the initial range, determined by the k-means clustering, we compare our grouping results with the ones that are obtained only using the hierarchical clustering. In order to compare the tract groups obtained using two different methods, we propose the following measure:

$$\begin{array}{c} Consistency\left(s_{i},s_{j}\right)=\frac{100}{n(s_{i})}\sum_{b_{i}\in s_{i}}\max_{b_{j}\in s_{j}}\frac{Count(b_{i}\cap b_{j})}{Count(b_{i})} \end{array}$$ (15)

where *n*(*s*_*i*_) denotes the number of groups in a subject *s*_*i*_, *b*_*i*_ is a tract group of *s*_*i*_, *b*_*i*_∩*b*_*j*_ is the intersection of *b*_*i*_ and *b*_*j*_, and *Count* is the number of tracts within a given group. For each hemisphere of every subject, we measured *Consistency*(*s*_*d*_, *s*_*o*_) and *Consistency*(*s*_*o*_, *s*_*d*_), where *s*_*d*_ represents the tract groups obtained directly using the hierarchical clustering, and *s*_*o*_ denotes those obtained using our grouping method.

#### Sensitivity and false discovery rate (FDR)

In order to measure the classification performance, we analyze the labeling results that are obtained through nested cross-validation. For this purpose, we define sensitivity and FDR. The sensitivity for an anatomic bundle is defined as follows:

$$\begin{array}{c} Sensitivity\left(B_{a}\right)=\frac{Count(B_{a}\cap B_{m})}{Count(B_{m})} \end{array}$$ (16)

where *B*_*m*_ denotes the set of tracts in a bundle that are labeled manually by experts, and *B*_*a*_ are the tracts for the same bundle that are labeled automatically by our approach. Assuming that the expert-labeled tracts are the ground-truth, the sensitivity measures the ratio of the ground-truth tracts in a bundle that are identified by our approach to all ground-truth tracts in it. The FDR is defined as follows:

$$\begin{array}{c} FDR\left(B_{a}\right)=1.0-\frac{Count(B_{a}\cap B_{m})}{Count(B_{a})} \end{array}$$ (17)

This FDR measures the ratio of the tracts in *B*_*a*_ that do not belong to *B*_*m*_ to all the tracts in it.

#### Kappa

For examining the agreement between two bundles, we adopt the kappa analysis as in [21]. The kappa value is commonly used for evaluating agreement between two raters, which is known to be robust since the kappa takes into account agreement by chance. Landis and Koch assigned labels to kappa value ranges as follows [55]: *κ* value smaller than 0 is “poor”, 0.00-0.20 is “slight”, 0.21-0.40 is “fair”, 0.41-0.60 is “moderate”, 0.61-0.80 is “substantial”, and 0.81-1.00 is “almost perfect” agreement. Each bundle is first converted to a binary image with the same dimension as diffusion-weighted images of example subjects (128 × 128 × 60). A pixel value in the binary image is set to one if any tract of a bundle passes through the pixel, and set to zero otherwise. Two binary images are superimposed to classify each pixel into three categories: pixels whose values are one in the both images (pp), pixels whose values are zero in the both images (nn), and pixels whose values are different in the two images (pn, np). Then, a probability of observed agreement (*p*_*o*_) and a probability of chance agreement (*p*_*e*_) are computed as follows:

$$\begin{array}{c} p_{o}=\frac{pp+nn}{N} \end{array}$$ (18)

$$\begin{array}{c} p_{e}=(\frac{pp+pn}{N})\cdot (\frac{pp+np}{N})+(\frac{nn+np}{N})\cdot (\frac{nn+pn}{N}) \end{array}$$ (19)

where *N* = *pp* + *nn* + *pn* + *np*. The pixels with FA values lower than 0.2 are not included in the computation. Finally, the kappa value *κ* for the two bundles is computed as follows:

$$\begin{array}{c} \kappa =\frac{p_{o}-p_{e}}{1-p_{e}} \end{array}$$ (20)

## Results and Discussion

Our approach for the automatic classification of WM tracts was validated through five experiments: nested cross-validation, comparison with a ROI-based labeling method [21], comparison with a Guevara et al.’s method, and advantage of the proposed multi-atlas approach. All experiments were performed on a PC equipped with an Intel Core i7 CPU, a NVIDIA GeForce GTX460 GPU card, and 32GB of RAM memory. We implemented the proposed approach using C++ and CUDA C, and the executable file will be made publicly available. In this section, we first discuss the choice of parameters for example data construction and then describe each experiment in detail.

### Parameter setting

We used twelve subjects of the NMR public database, as the example subjects (see MRI acquisition and preprocessing section), and we reconstructed the tracts by employing a tractography algorithm [43]. The average numbers of tracts for the left and right hemispheres of an example subject were 273,906 and 265,775, respectively. Each of the resulting tracts was resampled in order to use more effectively the computing power of the GPU. Specifically, we set the number of sample points for a tract to 32, since is more efficient to set the number of threads in a block as a multiples of 32 [56].

The tracts were first classified into 100 length range groups, according to their chord lengths through k-means clustering, and then tracts within each length range group were clustered according to their shape similarity by employing hierarchical clustering with average-linkage [57]. As will be shown in the Section Consistency of the tract grouping method, we verified that the value of 100 for length range groups gives almost similar results with conventional clustering method.

The distance threshold for hierarchical clustering was set to 40. Assuming that each pair of the corresponding sample points of two tracts has a constant distance, the distance 40 means that the distance between every pair of corresponding sample points is about 7mm. To merge similar groups in consecutive length ranges, we set the distance threshold value more tightly to 20, which indicates that the distance between the corresponding sample points of two mean curves is about 3.5mm. The above two distance thresholds were set empirically. However, the selected values could be used for other data sets if the parameters for DTI scanning protocol are similar to ours (e.g., voxel size), because those values are based on the actual fiber distance in millimeter.

The threshold value for identifying outlier groups was determined such that the sum of tracts in groups with number of tracts smaller than the threshold is within 2% of total number of tracts in a subject. In our tract grouping method, we tried to reassign tracts in each outlier group. The outlier tracts were identified by setting the threshold to the critical value for the 2% level in the Chi-square distribution with 96 dimensions. We internally tested with a range of values for above outlier thresholds, and found that similar grouping results could be obtained. The average number of resulting tract groups for the left and right hemispheres of an example subject were 2024 and 1970, respectively. The maximum number of tracts in an outlier group was 14 for both the left and right hemispheres.

### Consistency of the tract grouping method

Since the number of tracts in a subject is huge, the tracts can not be grouped directly using the conventional hierarchical clustering method. Thus, we first randomly chose 10,000 tracts in each hemisphere of every subject. The tracts of each hemisphere in a subject were then grouped using the two grouping methods: the proposed method and the hierarchical clustering method. The distance threshold for direct hierarchical clustering was set to 40. For our method, the number of length ranges was set to 100, and the distance thresholds for hierarchical clustering and for merging similar groups were both set to 40, for fair comparison. We did not perform the last outlier removal step. The average consistency for all subjects was 96.1%, which showed a high consistency between two methods.

### Nested cross-validation

**Fig 5:**
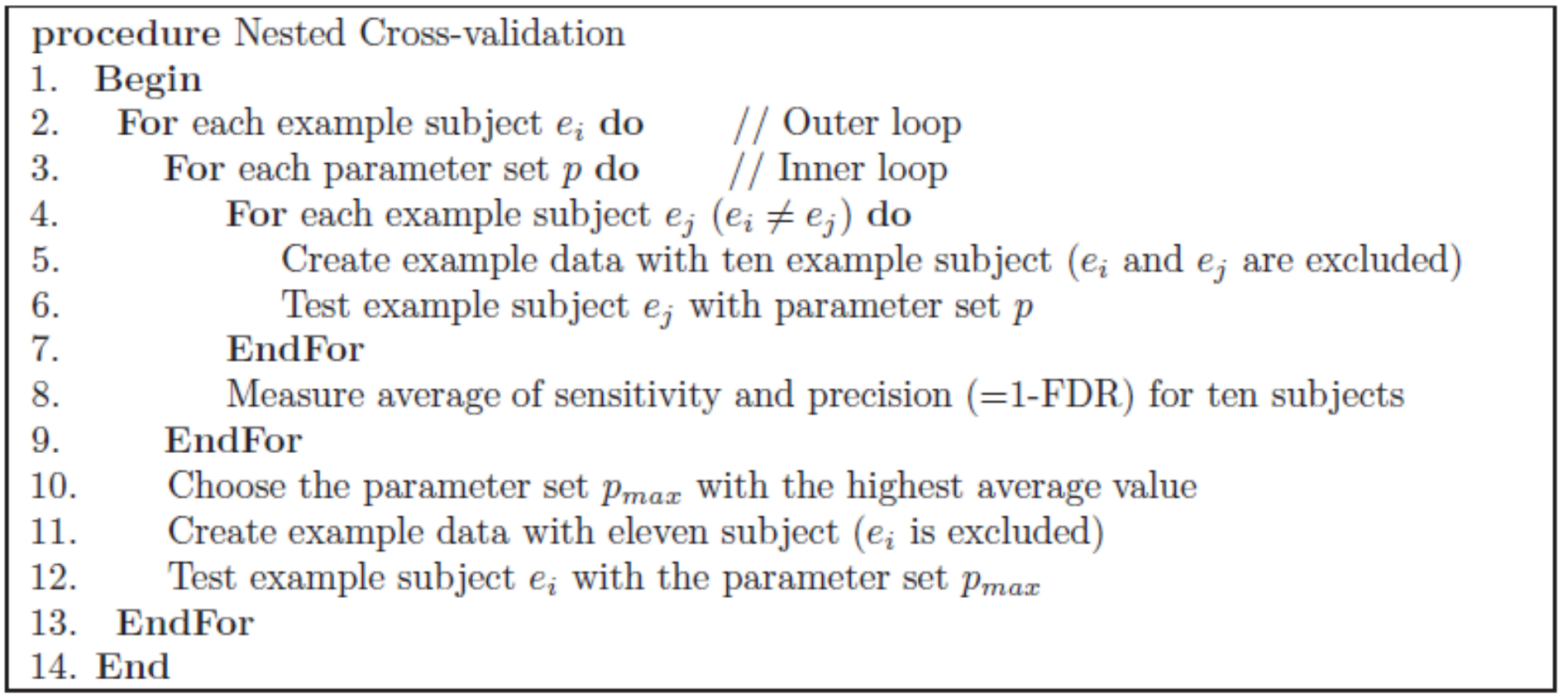
Skeleton of nested cross-validation. The skeleton of the nested cross-validation for measuring the performance of the proposed method.

The performance of the proposed approach for automatic classification was assessed through nested cross-validation. The nested cross-validation was used to avoid duplicate use of the same data for parameter tuning and testing. Fig 5 shows the skeleton of the nested cross-validation. The inner loop is used to determine the parameter set, while the outer loop is for testing each example subject data using the determined parameter set. The parameter set consists of distance threshold and vote number threshold. SKLD distance is used for the tract group labeling approach, while Mahalanobis distance was used for the direct tract labeling approach. We uniformly sampled between 40,000 and 60,000 for the SKLD distance threshold, and between 200 and 400 for the Mahalanobis distance threshold. The vote number threshold was uniformly sampled between 5 and 7.

The proposed approach took 4,688.4 seconds on average using only the CPU, and 19.0 seconds on average using both the CPU and GPU. Thus, our parallel labeling approach was about 246.8 times faster than its sequential version for the example data. The direct tract labeling approach took 53.1 seconds on average using the GPU version, which was about 2.8 times slower than the tract grouping approach. However, the direct tract labeling approach does not need to perform the tract grouping.

**Fig 6:**
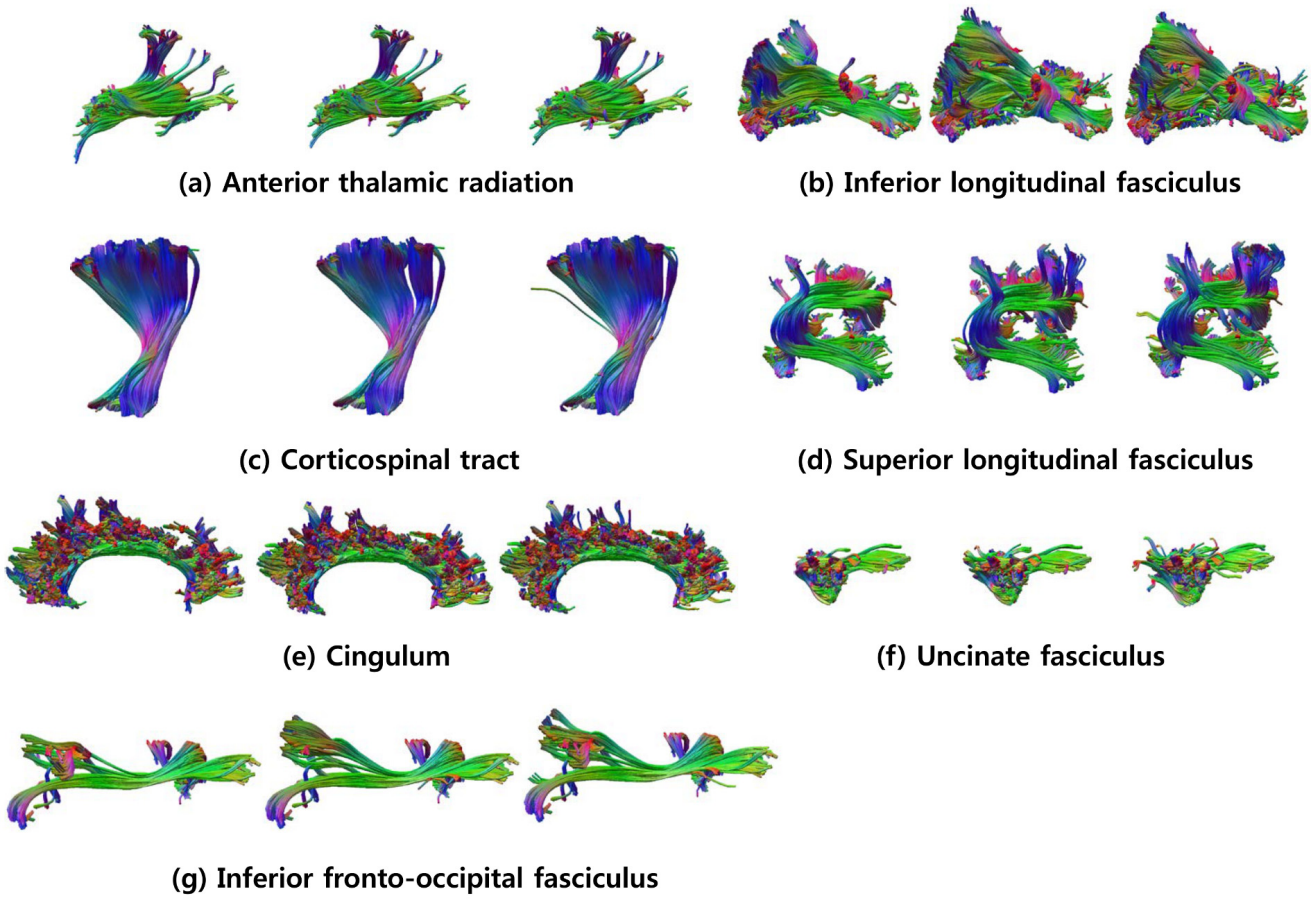
Comparison of expert-labeled bundles and automatically obtained bundles using our classification algorithm. The bundles in the first and fourth columns represent expert-labeled bundles, those in the second and fifth columns represent bundles obtained using tract group labeling, and those in the third and sixth columns represent bundles obtained using direct tract labeling.

Fig 6 illustrates the automatic labeling results of tracts for an example subject. Despite the variability of bundle shapes across different subjects, the overall shape of the automatically labeled tracts for every bundle were similar to that of the expert-labeled tracts, which visually validates our approach.

**Fig 7:**
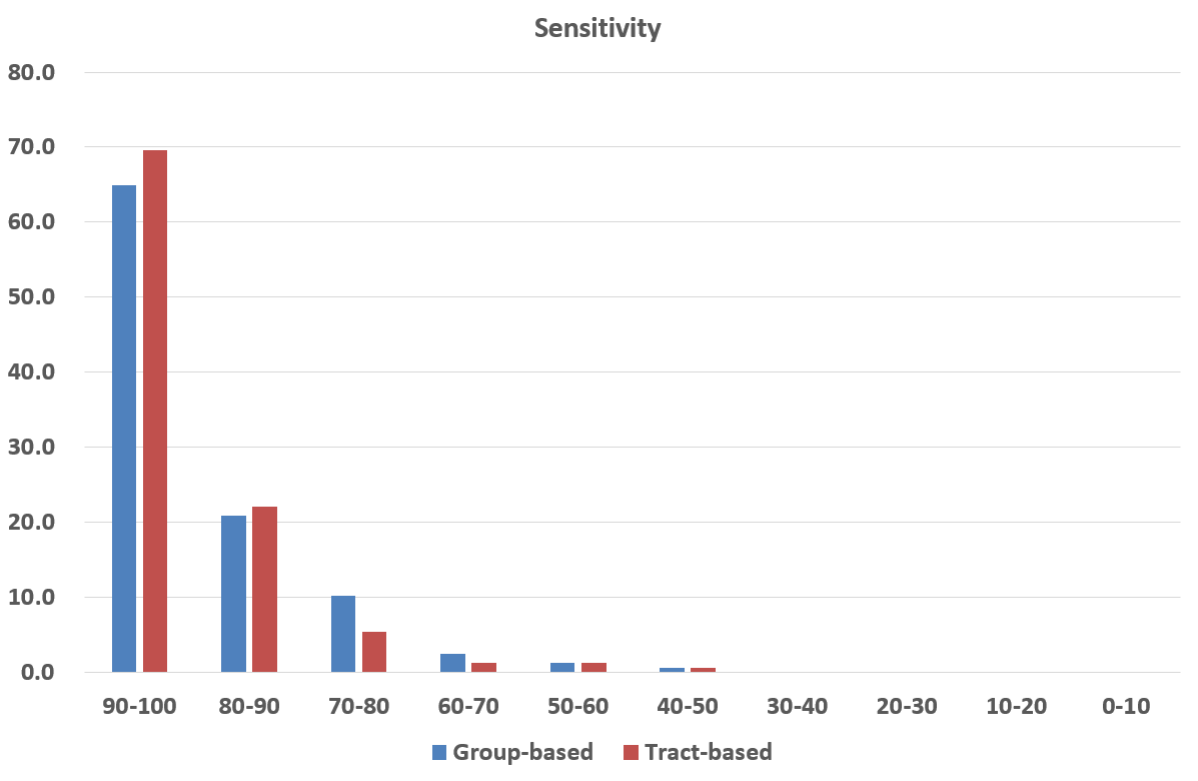
Sensitivity histogram for tract group and direct tract labeling. The x-axis and y-axis represents sensitivity ranges and percentage of bundles that are included in the corresponding sensitivity ranges, respectively.

Fig 7 presents the sensitivity of the tract group and direct tract labeling. The horizontal axis in the figure represents sensitivity ranges, and the vertical axis gives the percentage of bundles in each sensitivity range. The average sensitivity of bundles for the group labeling was 90.3% (LH: 89.5%, RH: 91.0%), while the value for the direct tract labeling was 91.9% (LH: 91.7%, RH: 92.1%). As shown in the results, the sensitivity of direct tract labeling was 1.6% higher than that of tract group labeling.

**Fig 8:**
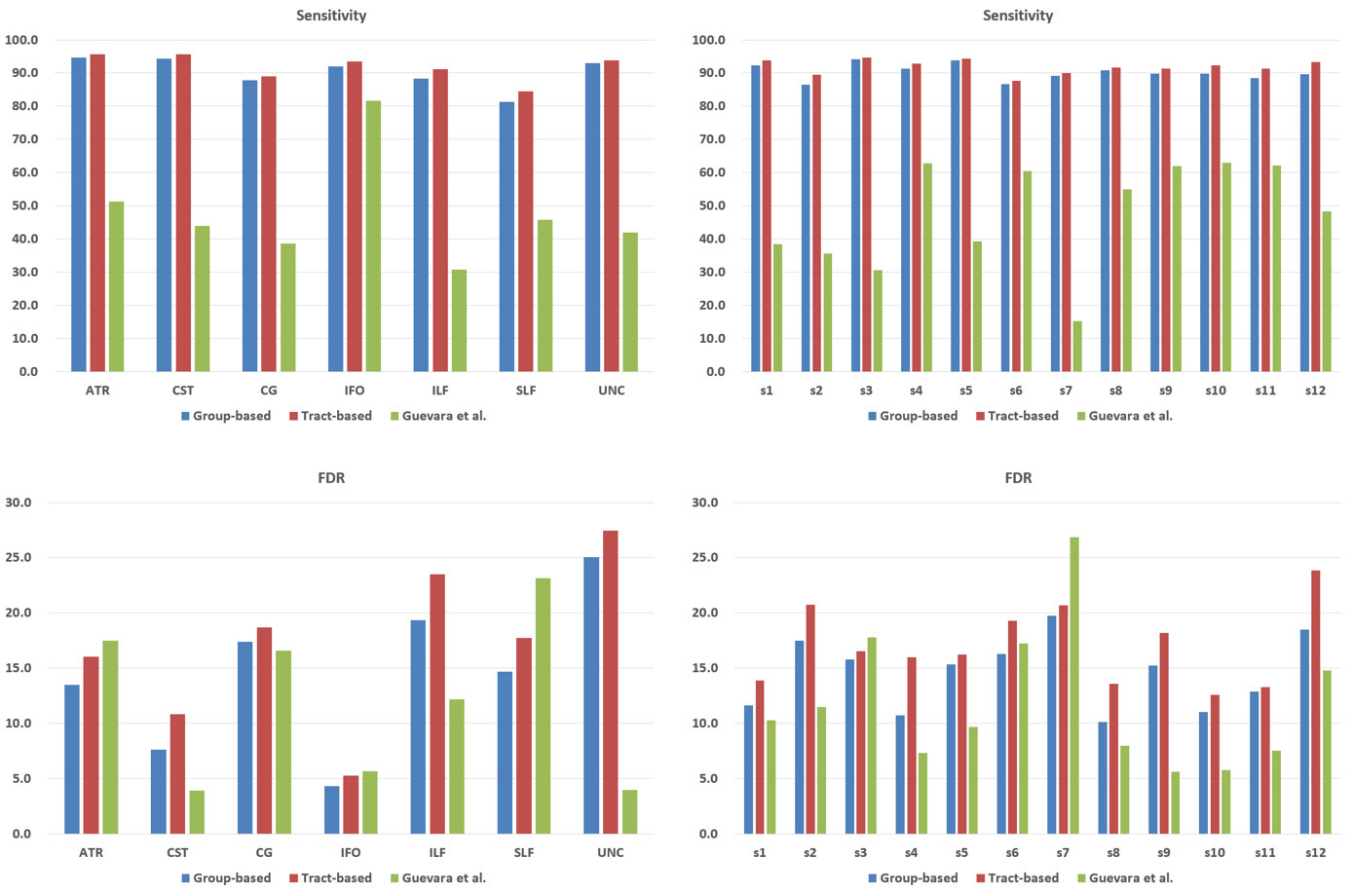
Sensitivity and FDR histograms for tract group labeling, direct tract labeling, and Guevara’s method. Top left: sensitivities of the three methods for each anatomic bundle, Top right: sensitivities of the three methods for each example subject. Bottom left: FDRs of the three methods for each anatomic bundle, Bottom right: FDRs of the three methods for each example subject.

Fig 8 illustrates the sensitivity and FDR of the tract group labeling and direct tract labeling. (Results for the Guevara et al.’s method will be described in Section Comparison with a Guevara et al.’s method.) The histograms in the left column shows that high sensitivities were observed for most anatomic bundles. For the tract group labeling, the sensitivities of the left/right anterior thalamic radiation (ATR), left/right corticospinal tract (CST), right inferior fronto-occipital fasciculus (IFO), right inferior longitudinal fasciculus (ILF), and left/right uncinate fasciculus (UNC) were more than 90%. The majority of anatomic bundles showed FDRs below 20%. Relatively high FDRs (more than 20%) for the ILF(L) and UNC(L,R) are due to their complex shapes and high shape variability across the example subjects. For the majority of subjects, high sensitivities (more than 90%) and low FDRs (less than 16%) were observed. The sensitivity of the direct group labeling was higher than that of tract grouping results, while the precision (=1-FDR) was lower: Specifically, the average sensitivity of tract group labeling results was 90.2% (LH: 89.5%, RH: 91.0%), while the average sensitivity of direct tract labeling results was 91.9% (LH: 91.7%, RH: 92.1%). Also, the average FDR of tract group labeling results was 14.6% (LH: 14.9%, RH: 14.2%), while the average FDR of direct tract labeling results was 17.1% (LH: 17.7%, RH: 16.4%). The group-based method showed smaller sensitivity and FDR because every tracts in a group are simultaneously labeled or considered as outlier. If some tracts in a group have different shapes from the ground-truth, then every tract in the group will have more chance to be labeled as outlier even if other tracts are similar to the ground-truth. We think that these situations could happen more if a tight distance threshold is used. Additionally, the FDR of the group-based result could be lower because the outliers are already removed in the tract grouping stage.

To validate our approach, we further measured FA and MD values for the seven anatomic bundles of twelve subjects, and compared those values between manually and automatically labeled bundles. Specifically, we computed a ratio of the FA(MD) difference to the FA(MD) of manually labeled bundles. Table 2 shows the results: The ratio were all less than 4.1%. The bundle with the highest difference in the ratio was UNC, and we speculate that the reason is its relatively high FDR and small number of tracts within it.

**Table 2:** Comparison of diffusion properties (FA,MD) between manually and automatically labeled bundles.

	ATR	CST	CG	IFO	ILF	SLF	UNC
FA (LH)	2.1%	1.2%	1.7%	0.6%	1.4%	0.6%	2.3%
FA (RH)	1.9%	1.0%	1.2%	0.4%	0.9%	2.1%	4.1%
MD (LH)	0.2%	0.6%	0.7%	0.4%	0.4%	0.3%	1.4%
MD (RH)	0.3%	0.6%	0.7%	0.3%	0.3%	0.7%	1.7%

Comparison is done by computing the ratio of the FA(MD) difference to the FA(MD) of manually labeled bundles. The ratio is averaged over the values of twelve subjects.

### Comparison with a ROI-based method

We compared our approach with a ROI-based labeling method [21]. In the latter method, a set of ROIs for a test subject were automatically obtained by non-linearly warping an atlas with 130 ROIs to the test subject data using the LDDMM [58]. Based on these ROIs and anatomic knowledge on ROIs through which each bundle passes, 30 prominent and well-known bundles were extracted.

For comparison, we focused on six anatomic bundles that were commonly handled in the ROI-based method and our approach. Specifically, we obtained six anatomic bundles for each hemisphere of twelve example subjects by using their method and our approach, and compared expert-labeled bundles with bundles acquired using the ROI-based method and also with those obtained using our approach. To segment bundles of an example subject using our approach, the expert-labeled bundles for the other eleven example subjects were used as example data.

We computed the average and standard deviation of kappa values for each bundle from twelve example subjects. In Table 3, the first row shows the kappa statistics computed between expert-labeled bundles and bundles obtained using our approach, while the second row exhibits those between expert-labeled bundles and bundles acquired using the method of Zhang et al. [21]. The average kappa values of bundles obtained using our approach were 0.87 and 0.88 for the left and right hemispheres, respectively, and those for bundles acquired using their method were 0.59 and 0.60, respectively. For every bundle, our approach showed better accuracy than the ROI-based automatic labeling method [21] in terms of kappa values. It is worth noting that our method is not biased to the expert example data because we used the nested cross-validation for parameter tuning.

**Table 3:** Comparison of kappa statistics with a ROI-based method [21].

		CST	CG	IFO	ILF	SLF	UNC
Ours	Left	0.89±0.07	0.79±0.10	0.90±0.07	0.85±0.05	0.89±0.03	0.89±0.06
	Right	0.91±0.05	0.81±0.06	0.93±0.06	0.89±0.04	0.86±0.05	0.89±0.13
Zhang et al. [21]	Left	0.29±0.14	0.73±0.08	0.74±0.12	0.57±0.08	0.71±0.08	0.52±0.18
	Right	0.36±0.12	0.69±0.07	0.73±0.10	0.53±0.05	0.68±0.07	0.63±0.17

The average and standard deviation of kappa values are computed for each bundle from twelve example subjects. The first row shows the kappa statistics between expert-labeled bundles and bundles obtained using our approach, and the second row presents those between expert-labeled bundles and bundles acquired using method of Zhang et al. [21].

**Fig 9:**
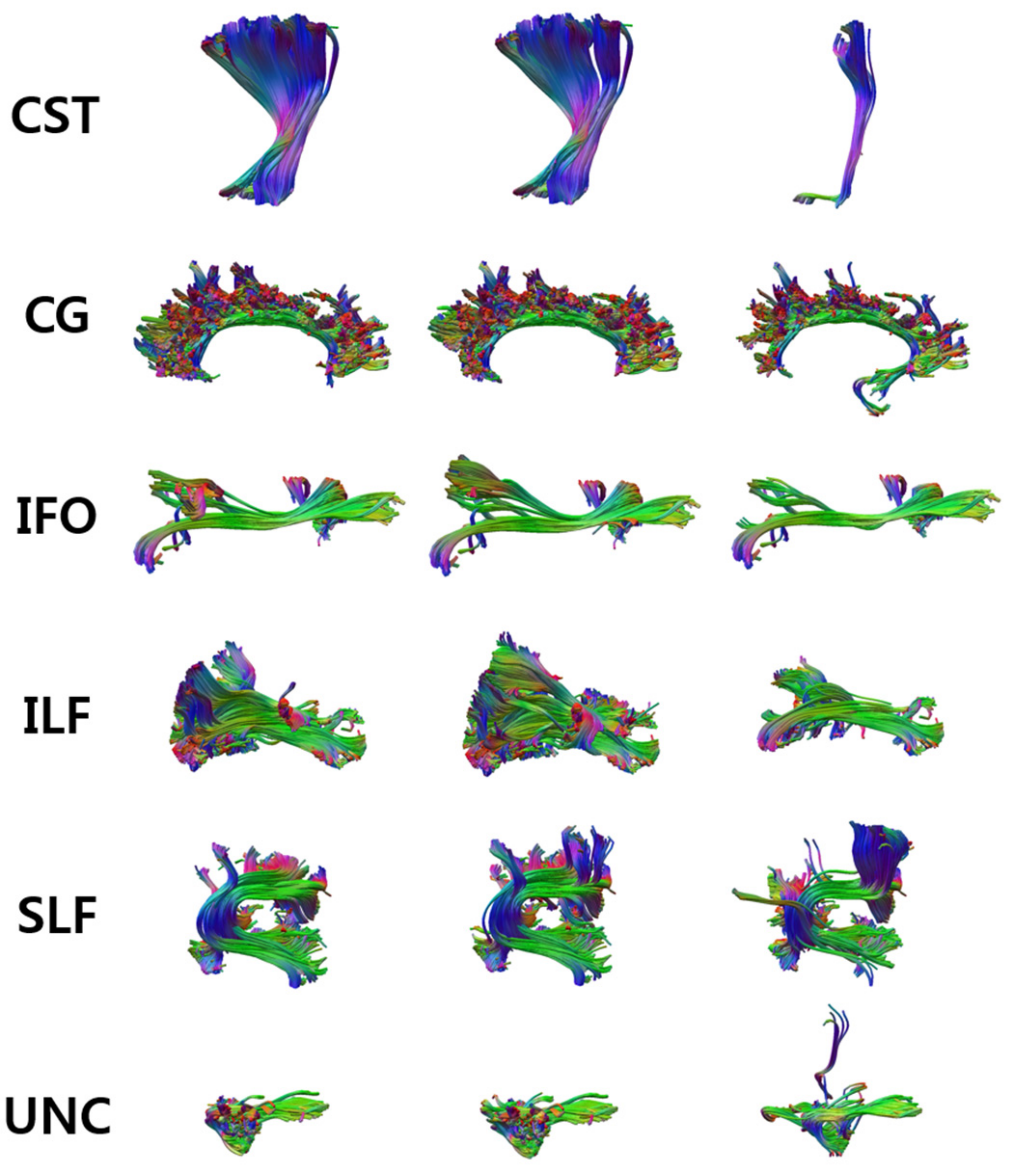
Comparison with a ROI-based method [21]. The left column represents expert-labeled bundles, the middle column depicts bundles obtained using our approach, and the right column shows bundles acquired using the ROI-based method. Our approach obtained more accurate labeling results than Zhang et al.’s method [21] by modeling the shape of tracts mathematically.

In Fig 9, the left column presents the expert-labeled bundles of an example subject, the middle column exhibits bundles obtained using our approach, and the right column shows bundles acquired using method of Zhang et al. [21]. By comparing the bundle shapes in the left and right columns, we can verify that the labeling results by our approach are more similar to the expert-provided results than the labeling results from the ROI-based method. For CG and UNC, the labeling results of the latter method show tracts which are quite different in their shape from those of the expert-provided results. This shape difference may result from the registration error for obtaining ROIs. WM tracts are directly modeled in our approach based on their shapes and trajectories without using ROIs, which yields more similar results to the expert-labeled tracts for all bundles.

### Comparison with a Guevara et al.’s method

We compared our approach with a Guevara et al.’s method [33]. For comparison, we measured sensitivity and FDR for seven bundles of twelve subjects. We used the same input data, but different example data set.

**Fig 10:**
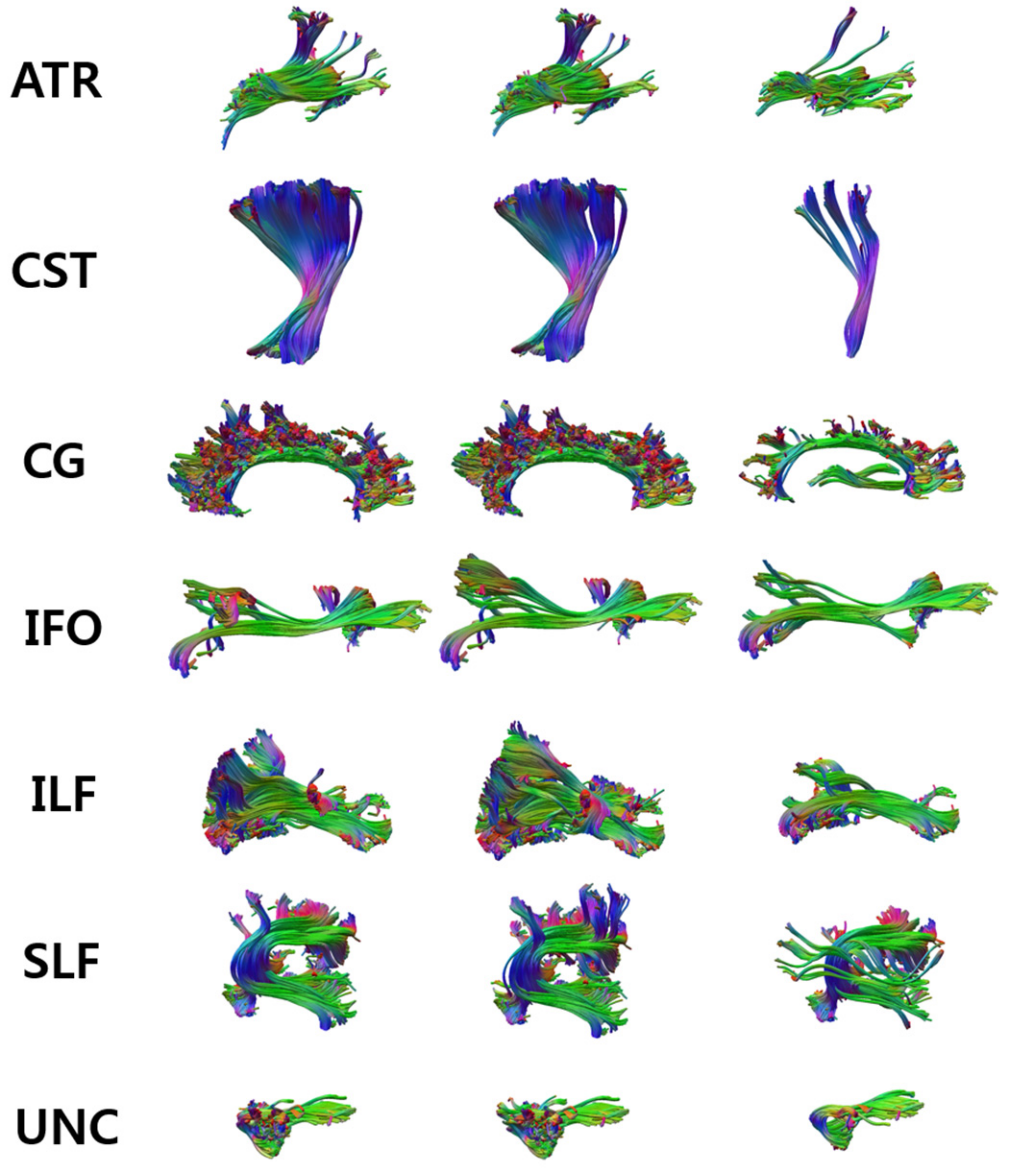
Comparison with a Guevara et al.’s method [33]. The left column represents expert-labeled bundles, the middle column depicts bundles obtained using our approach, and the right column shows bundles acquired using the Guevara et al.’s method. Our approach obtained more accurate labeling results than Guevara et al.’s method [33].

In Fig 10, the left column presents the expert-labeled bundles of an example subject, the middle column exhibits bundles obtained using our approach, and the right column shows bundles acquired using the method of Guevara et al. [33]. By comparing the bundle shapes in the left and right columns, we can verify that the labeling results by our approach are more similar to the expert-provided results than the labeling results from the Guevara et al.’s method. For CG and SLF, the labeling results of the latter method contain a few outlier tracts. These outliers may result from accidental matching of outliers with example data. Our method can reduce this type of labeling error by using the voting scheme.

Fig 8 shows the sensitivity of the Guevara et al.’s method and its FDR. The left column shows the sensitivity and FDR for each anatomic bundle, and the right column exhibits the same measures for each example subject. The average sensitivity was 47.7% (LH: 50.1%, RH: 45.3%), and the FDR was 11.9% (LH: 11.3%, RH: 12.4%). We speculate that the low sensitivity values compared to our methods are due to the fact that the atlas used in the Guevara et al.’s method contains only long tracts, while our atlas includes short tracts as well as long ones.

### Advantage of the proposed multi-atlas approach

**Fig 11:**
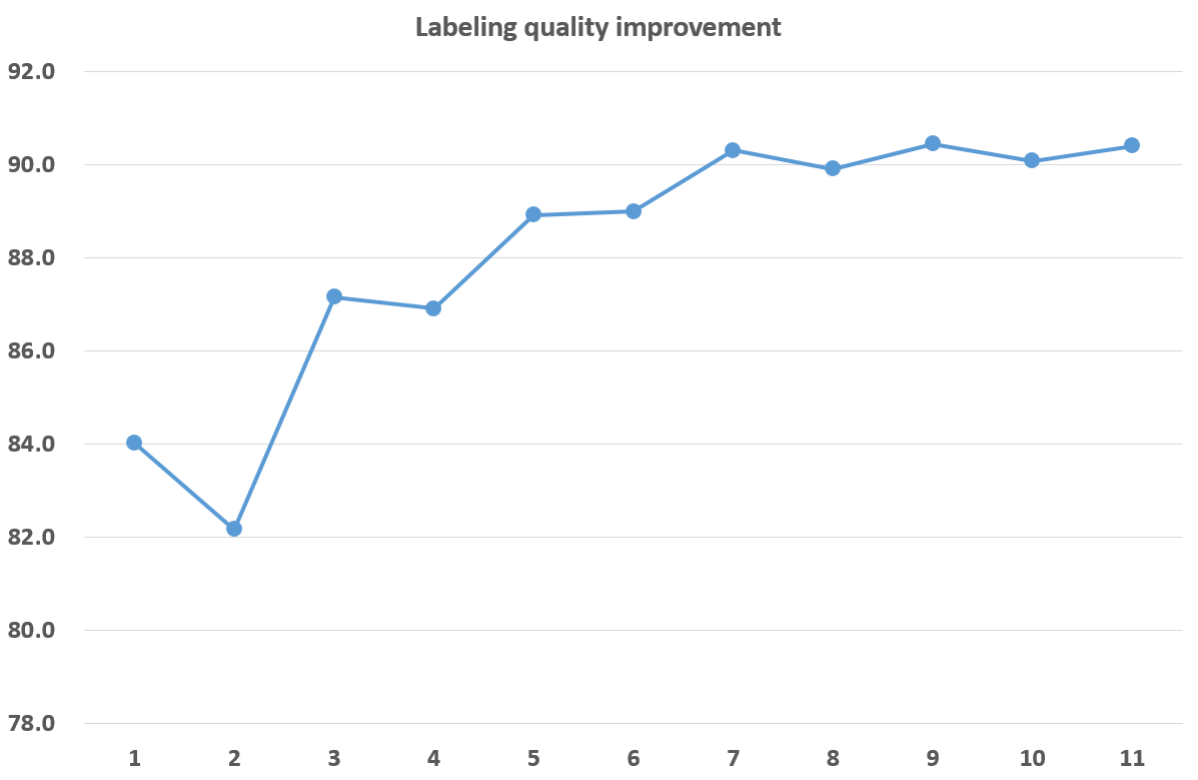
Labeling quality improvement. Change of labeling quality as the number of example subjects increases from one to eleven.

To demonstrate the benefit of our multi-atlas approach, we measured the performance by adding an example subject incrementally. Specifically, we performed our method to label tracts in one randomly chosen subject while increasing the number of example subjects from one to eleven. The labeling performance was measured as the average of sensitivity and precision for every bundle. The SKLD threshold value was set to 50,000, and the threshold value for the maximum number of votes was set to majority of the number of example subjects. Fig 11 shows the results: The performance was improved by 6.4% (LH: 4.1%, RH: 8.6%) when the number of example subjects was increased from one to eleven.

## Conclusions

In this paper, we presented an example-based multi-atlas approach for labeling the WM tracts of a test subject. To construct the example data, experts manually labeled tracts of twelve example subjects based on their neuroanatomic knowledge. Multiple atlases were adopted to model the example data from these subjects, allowing the addition and deletion of subject example data in a flexible way. A voting scheme was proposed to facilitate the multi-atlas representation of example data, which was also effective for removing outliers. Our approach was simple and easy to implement by adopting the same metrics in both example data construction and tract labeling. For labeling efficiency, the tracts of a test subject were classified into groups according to their shape similarity, and the tracts within each group were labeled simultaneously by exploiting the expert-labeled example data. The resulting labels of input tracts reflected well the neuroanatomical convention conveyed by the example data. We further accelerated our approach by implementing it on the GPU. Through nested cross-validation, we demonstrated high performance in terms of the sensitivity and FDR.

**Fig 12:**
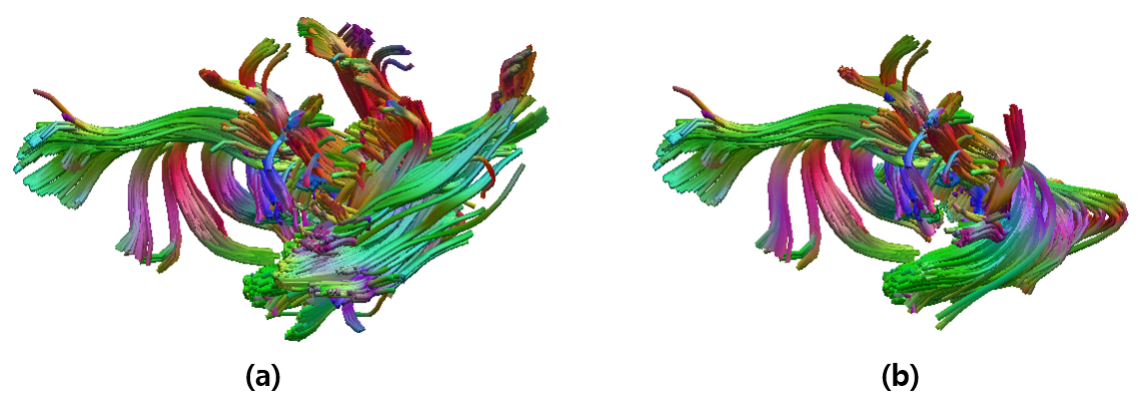
Limitation of the proposed approach. Automatically labeled UNC bundle in (a) shows low sensitivity (79.4%) and high FDR (51.2%) values due to its high shape variability. The bundle in (b) demonstrates the manually labeled UNC bundle.

Our approach showed high sensitivity and low FDR values for most of the bundles, but some bundles showed relatively low sensitivity and high FDR values because of their high shape variability, which were UNC and ILF. Fig 12 shows one example of automatically labeled bundles with low performance. In order to increase the performance, we are planning to incorporate the connectivity information together with the currently used bundle shape information when labeling the tracts.

Although we used seven anatomic bundles listed in Table 1, different types of bundles were used in general for diagnosis of different brain diseases. We are planning to investigate the difference in diffusion properties such as FA and MD between normal groups and patient groups for neurological diseases such as multiple sclerosis or Alzheimer’s disease, by dealing with bundles such as corpus callosum and fornix on top of the seven bundles.

Although we classified input tracts into seven bundles which have been commonly used in previous work (see Table 1 for the list of the major bundles), we could also add other bundles such as external capsule (EC), short association bundles, commissural bundles, and U-shaped bundles. However, unlike the seven major bundles, these bundles are not consistent over all example subjects due to individual variability. Short association bundles have not been even completely anatomically identified yet. Our approach can handle non-identified bundles by assigning arbitrary labels to them, but their anatomical identification should be discussed through histological analysis in both human and nonhuman primates.

## Supporting Information

S1 Text GPU Implementation. (PDF)

S1 Fig The first stage of our parallel algorithm. In the first stage, the grid consists of *N* × *M* blocks, and each block contains *k* threads. A block (*i*, *j*) is used for computing the SKLD between an input tract group *g*_*i*_ and an example tract group *b*_*j*_. The resulting SKLDs are stored in an *N* × *M* array in the GPU memory.
(TIF)

S2 Fig The second stage of our parallel algorithm. In the second stage, the grid consists of *N* blocks, and each block contains *T* threads. The threads in a block are used to label an input group *g*_*i*_ based on voting scheme. The resulting labels are stored in the GPU memory space of size *N*.
(TIF)

## References

[1] Conturo TE, Lori NF, Cull TS, Akbudak E, Snyder AZ, Shimony JS, et al Tracking neuronal fiber pathways in the living human brain. Proceedings of the National Academy of Sciences of the United States of America. 1999;96(18):10422–10427. 10.1073/pnas.96.18.10422 10468624 PMC17904

[2] Mori S, Crain BJ, Chacko VP, van Zijl PC. Three-dimensional tracking of axonal projections in the brain by magnetic resonance imaging. Annals of neurology. 1999 2;45(2):265–269. 10.1002/1531-8249(199902)45:2<265::AID-ANA21>3.0.CO;2-3 9989633

[3] Basser PJ, Pajevic S, Pierpaoli C, Duda J, Aldroubi A. In vivo fiber tractography using DT-MRI data. Magnetic resonance in medicine: official journal of the Society of Magnetic Resonance in Medicine / Society of Magnetic Resonance in Medicine. 2000 10;44(4):625–632. 10.1002/1522-2594(200010)44:4<625::AID-MRM17>3.0.CO;2-O 11025519

[4] Koch MA, Norris DG, Hund-Georgiadis M. An Investigation of Functional and Anatomical Connectivity Using Magnetic Resonance Imaging. NeuroImage. 2002;16(1):241–250. 10.1006/nimg.2001.1052 11969331

[5] Descoteaux M, Deriche R, Knosche TR, Anwander A. Deterministic and Probabilistic Tractography Based on Complex Fibre Orientation Distributions. Medical Imaging, IEEE Transactions on. 2009 2;28(2):269–286. 10.1109/TMI.2008.2004424 19188114

[6] Wakana S, Jiang H, Nagae-Poetscher LM, van Zijl PCM, Mori S. Fiber Tract-based Atlas of Human White Matter Anatomy. Radiology. 2004;230(1):77–87. 10.1148/radiol.2301021640 14645885

[7] Glenn OA, Henry RG, Berman JI, Chang PC, Miller SP, Vigneron DB, et al DTI-based three-dimensional tractography detects differences in the pyramidal tracts of infants and children with congenital hemiparesis. Journal of Magnetic Resonance Imaging. 2003;18(6):641–648. 10.1002/jmri.10420 14635148

[8] Goldberg-Zimring D, Mewes AU, Maddah M, Warfield SK. Diffusion tensor magnetic resonance imaging in multiple sclerosis. J Neuroimaging. 2005;15(4 Suppl):68S–81S. 10.1177/1051228405283363 16385020

[9] Pagani E, Filippi M, Rocca MA, Horsfield MA. A method for obtaining tract-specific diffusion tensor MRI measurements in the presence of disease: application to patients with clinically isolated syndromes suggestive of multiple sclerosis. NeuroImage. 2005;26(1):258–265. 10.1016/j.neuroimage.2005.01.008 15862226

[10] Kubicki M, McCarley R, Westin CF, Park HJ, Maier S, Kikinis R, et al A review of diffusion tensor imaging studies in schizophrenia. Journal of Psychiatric Research. 2007;41(1–2):15–30. 10.1016/j.jpsychires.2005.05.005 16023676 PMC2768134

[11] Ciccarelli O, Catani M, Johansen-Berg H, Clark C, Thompson A. Diffusion-based tractography in neurological disorders: concepts, applications, and future developments. The Lancet Neurology. 2008;7(8):715–727. 10.1016/S1474-4422(08)70163-7 18635020

[12] Hua K, Zhang J, Wakana S, Jiang H, Li X, Reich DS, et al Tract probability maps in stereotaxic spaces: Analyses of white matter anatomy and tract-specific quantification. NeuroImage. 2008;39(1):336–347. 10.1016/j.neuroimage.2007.07.053 17931890 PMC2724595

[13] Berman JI, Mukherjee P, Partridge SC, Miller SP, Ferriero DM, Barkovich AJ, et al Quantitative diffusion tensor MRI fiber tractography of sensorimotor white matter development in premature infants. NeuroImage. 2005;27(4):862–871. 10.1016/j.neuroimage.2005.05.018 15978841

[14] Goodlett CB, Fletcher PT, Gilmore JH, Gerig G. Group analysis of DTI fiber tract statistics with application to neurodevelopment. NeuroImage. 2009;45(1, Supplement 1):S133—S142. Mathematics in Brain Imaging. 10.1016/j.neuroimage.2008.10.060 19059345 PMC2727755

[15] Maddah M, Grimson WEL, Warfield SK, Wells WM. A unified framework for clustering and quantitative analysis of white matter fiber tracts. Medical Image Analysis. 2008;12(2):191–202. 10.1016/j.media.2007.10.003 18180197 PMC2615202

[16] Li H, Xue Z, Guo L, Liu T, Hunter J, Wong STC. A hybrid approach to automatic clustering of white matter fibers. NeuroImage. 2010;49(2):1249–1258. 10.1016/j.neuroimage.2009.08.017 19683061

[17] Catani M, Howard RJ, Pajevic S, Jones DK. Virtual in Vivo Interactive Dissection of White Matter Fasciculi in the Human Brain. NeuroImage. 2002;17(1):77–94. 10.1006/nimg.2002.1136 12482069

[18] Wakana S, Caprihan A, Panzenboeck MM, Fallon JH, Perry M, Gollub RL, et al Reproducibility of quantitative tractography methods applied to cerebral white matter. NeuroImage. 2007;36(3):630–644. 10.1016/j.neuroimage.2007.02.049 17481925 PMC2350213

[19] Catani M, de Schotten MT. A diffusion tensor imaging tractography atlas for virtual in vivo dissections. Cortex. 2008;44(8):1105–1132. Special Issue on “Brain Hodology—Revisiting disconnection approaches to disorders of cognitive function”. 10.1016/j.cortex.2008.05.004 18619589

[20] Wassermann D, Bloy L, Kanterakis E, Verma R, Deriche R. Unsupervised white matter fiber clustering and tract probability map generation: Applications of a Gaussian process framework for white matter fibers. NeuroImage. 2010;51(1):228–241. 10.1016/j.neuroimage.2010.01.004 20079439 PMC2847030

[21] Zhang Y, Zhang J, Oishi K, Faria AV, Jiang H, Li X, et al Atlas-guided tract reconstruction for automated and comprehensive examination of the white matter anatomy. NeuroImage. 2010;52(4):1289–1301. 10.1016/j.neuroimage.2010.05.049 20570617 PMC2910162

[22] Bazin PL, Ye C, Bogovic JA, Shiee N, Reich DS, Prince JL, et al Direct segmentation of the major white matter tracts in diffusion tensor images. NeuroImage. 2011;58(2):458–468. 10.1016/j.neuroimage.2011.06.020 21718790 PMC3159825

[23] O’Donnell L, Kubicki M, Shenton ME, Dreusicke MH, Grimson WEL, Westin CF. A method for clustering white matter fiber tracts. American Journal of Neuroradiology. 2006;27(5):1032–1036. 16687538 PMC2768142

[24] Guevara P, Cointepas Y, Rivière D, Poupon C, Thirion B, Mangin JF. Inference of a fiber bundle atlas using a two-level clustering strategy. In: MICCAI 2009 Workshop on Diffusion Modelling; 2009. 10.1007/978-3-642-15705-9_67 20879274

[25] Guevara P, Poupon C, Rivière D, Cointepas Y, Descoteaux M, Thirion B, et al Robust clustering of massive tractography datasets. NeuroImage. 2011;54(3):1975–1993. 10.1016/j.neuroimage.2010.10.028 20965259

[26] Wang X, Grimson WEL, Westin CF. Tractography segmentation using a hierarchical Dirichlet processes mixture model. NeuroImage. 2011;54(1):290–302. 10.1016/j.neuroimage.2010.07.050 20678578 PMC2962770

[27] Visser E, Nijhuis EH, Buitelaar JK, Zwiers MP. Partition-based mass clustering of tractography streamlines. Neuroimage. 2011;54(1):303–312. 10.1016/j.neuroimage.2010.07.038 20673849

[28] Wu X, Xie M, Zhou J, Anderson AW, Gore JC, Ding Z. Globally optimized fiber tracking and hierarchical clustering—a unified framework. Magnetic resonance imaging. 2012;30(4):485–495. 10.1016/j.mri.2011.12.017 22285879 PMC3327795

[29] Dodero L, Vascon S, Murino V, Bifone A, Gozzi A, Sona D. Automated multi-subject fiber clustering of mouse brain using dominant sets. Frontiers in Neuroinformatics. 2015;8:87 10.3389/fninf.2014.00087 25628561 PMC4290731

[30] Shi J, Malik J. Normalized Cuts and Image Segmentation. IEEE Trans Pattern Anal Mach Intell. 2000;22(8):888–905. 10.1109/34.868688

[31] O’Donnell LJ, Westin CF. Automatic Tractography Segmentation Using a High-Dimensional White Matter Atlas. IEEE Transactions on Medical Imaging. 2007 11;26(11):1562–1575. 10.1109/TMI.2007.906785 18041271

[32] Yendiki A, Panneck P, Srinivasan P, Stevens A, Zöllei L, Augustinack J, et al Automated probabilistic reconstruction of white-matter pathways in health and disease using an atlas of the underlying anatomy. Frontiers in neuroinformatics. 2011;5 10.3389/fninf.2011.00023 22016733 PMC3193073

[33] Guevara P, Duclap D, Poupon C, Marrakchi-Kacem L, Fillard P, Bihan DL, et al Automatic fiber bundle segmentation in massive tractography datasets using a multi-subject bundle atlas. NeuroImage. 2012;61(4):1083–1099. 10.1016/j.neuroimage.2012.02.071 22414992

[34] Wang Q, Yap PT, Wu G, Shen D. Application of neuroanatomical features to tractography clustering. Human brain mapping. 2013;34(9):2089–2102. 10.1002/hbm.22051 22461221 PMC4154701

[35] Jin Y, Shi Y, Zhan L, Gutman BA, de Zubicaray GI, McMahon KL, et al Automatic clustering of white matter fibers in brain diffusion MRI with an application to genetics. NeuroImage. 2014;100:75–90. 10.1016/j.neuroimage.2014.04.048 24821529 PMC4255631

[36] Tunç B, Parker WA, Ingalhalikar M, Verma R. Automated tract extraction via atlas based Adaptive Clustering. NeuroImage. 2014;102:596–607. 10.1016/j.neuroimage.2014.08.021 25134977 PMC4252913

[37] Fowlkes C, Belongie S, Chung F, Malik J. Spectral Grouping Using the Nyström Method. IEEE Trans Pattern Anal Mach Intell. 2004;26(2):214–225. 10.1109/TPAMI.2004.1262185 15376896

[38] Labra N, Figueroa M, Guevara P, Duclap D, Houenou J, Poupon C, et al. Interactive segmentation of white-matter fibers using a multi-subject atlas. In: Engineering in Medicine and Biology Society (EMBC), 2014 36th Annual International Conference of the IEEE. IEEE; 2014. p. 2376–2379. 10.1109/EMBC.2014.6944099 25570467

[39] Poupon C, Poupon F, Allirol L, Mangin JF. NMR: a free database dedicated to the anatomo-functional study of the human brain connectivity. Proceedings of the 12th Annual Meeting of the Organization for Human Brain Mapping. 2006;.

[40] Reese TG, Heid O, Weisskoff RM, Wedeen VJ. Reduction of eddy-current-induced distortion in diffusion MRI using a twice-refocused spin echo. Magnetic Resonance in Medicine. 2003;49(1):177–182. 10.1002/mrm.10308 12509835

[41] Descoteaux M, Angelino E, Fitzgibbons S, Deriche R. Regularized, fast, and robust analytical Q-ball imaging. Magn Reson Med. 2007 9;58(3):497–510. 10.1002/mrm.21277 17763358

[42] Goh A, Lenglet C, Thompson PM, Vidal R. Estimating Orientation Distribution Functions with Probability Density Constraints and Spatial Regularity. In: Proceedings of the 12th International Conference on Medical Image Computing and Computer-Assisted Intervention: Part I. MICCAI’09. Berlin, Heidelberg: Springer-Verlag; 2009. p. 877–885. 10.1007/978-3-642-04268-3_108 20426071

[43] Perrin M, Poupon C, Cointepas Y, Rieul B, Golestani N, Pallier C, et al. Fiber tracking in Q-ball fields using regularized particle trajectories. In: Proc. of IPMI; 2005. p. 52–63. 10.1007/11505730_5 17354684

[44] Perrin M, Cointepas Y, Cachia A, Poupon C, Thirion B, Riviere D, et al Connectivity-Based Parcellation of the Cortical Mantle Using q-Ball Diffusion Imaging. International Journal of Biomedical Imaging. 2008;2008(3):368406 10.1155/2008/368406 18401457 PMC2288697

[45] Smith SM, Jenkinson M, Woolrich MW, Beckmann CF, Behrens TEJ, Johansen-Berg H, et al Advances in functional and structural MR image analysis and implementation as FSL. NeuroImage. 2004;23, Supplement 1(0):S208—S219. 10.1016/j.neuroimage.2004.07.051 15501092

[46] O’Donnell LJ, Rigolo L, Norton I, III WMW, Westin CF, Golby AJ. fMRI-DTI modeling via landmark distance atlases for prediction and detection of fiber tracts. NeuroImage. 2012;60(1):456–470. 10.1016/j.neuroimage.2011.11.014 22155376 PMC3423975

[47] Schäfer J, Strimmer K. A Shrinkage Approach to Large-Scale Covariance Matrix Estimation and Implications for Functional Genomics. Statistical Applications in Genetics and Molecular Biology. 2005 11;4(1). 16646851 10.2202/1544-6115.1175

[48] Johnson S. Hierarchical clustering schemes. Psychometrika. 1967;32:241–254. 10.1007/BF02289588 5234703

[49] MacQueen JB. Some Methods for Classification and Analysis of MultiVariate Observations In: Cam LML, Neyman J, editors. Proc. of the fifth Berkeley Symposium on Mathematical Statistics and Probability. vol. 1 University of California Press; 1967 p. 281–297.

[50] Garyfallidis E, Brett M, Correia MM, Williams GB, Nimmo-Smith I. Quickbundles, a method for tractography simplification. Frontiers in neuroscience. 2012;6 10.3389/fnins.2012.00175 23248578 PMC3518823

[51] Duda RO, Hart PE, Stork DG. Pattern classification. 2nd. Edition New York 2001;.

[52] Santos-Pereira CM, Pires AM. Detection of outliers in multivariate data, a method based on clustering and robust estimators In: Proceedings in Computational Statistics. Physica-Verlag; 2002 p. 291–296.

[53] Kullback S, Leibler RA. On Information and Sufficiency. Ann Math Statist. 1951;22(1):79–86. 10.1214/aoms/1177729694

[54] Kullback S. An Application of Information Theory to Multivariate Analysis. The Annals of Mathematical Statistics. 1952;23(1):pp. 88–102. 10.1214/aoms/1177729487

[55] Landis JR, Koch GG. The Measurement of Observer Agreement for Categorical Data. Biometrics. 1977;33(1):pp. 159–174. 10.2307/2529310 843571

[56] Nvidia C. NVIDIA CUDA C Programming Guide. NVIDIA Corporation. 2012;Version 4.2:1–173.

[57] Sokal RR, Michener CD. A statistical method for evaluating systematic relationships. University of Kansas Scientific Bulletin. 1958;28:1409–1438.

[58] Ceritoglu C, Oishi K, Li X, Chou MC, Younes L, Albert M, et al Multi-contrast large deformation diffeomorphic metric mapping for diffusion tensor imaging. NeuroImage. 2009;47(2):618–627. 10.1016/j.neuroimage.2009.04.057 19398016 PMC2857762

