# Supplementary material for: An Example-Based Multi-Atlas Approach to Automatic Labeling of White Matter Tracts
Source: PLoS One. 2015 Jul 30;10(7):e0133337. doi: 10.1371/journal.pone.0133337 (PMC4520495; doi:10.1371/journal.pone.0133337)
Supplement: S1 Text — (PDF) [file pone.0133337.s001.pdf]

S1 Text. GPU implementation

In order to label all input WM tract groups, the SKLD between each of the input tract groups and every tract group in the example data should be computed. The time requirement for computing all SKLDs is very demanding with sequential computation. Given the input groups and the example groups, the SKLDs can be computed independently. Based on this observation, we compute the SKLDs in parallel for accelerating the input group labeling. We employ the Compute Unified Device Architecture (CUDA) of NVIDIA to facilitate parallel labeling. Threads in this architecture are organized hierarchically: a group of threads constitute a block, and blocks are aggregated to form a grid. A set of threads constituting a grid are executed together on the GPU.

Our parallel algorithm is given in Fig. 4. Due to the limitation on the maximum (allowed) number of blocks in a grid, a limited number of input groups are labeled simultaneously for each iteration of the outermost parallel loop (steps 2-16). The inner loop (steps 3-11) consists of two stages: In the first stage, the SKLDs are computed in parallel (steps 4-6). In the second stage, the resulting SKLDs are used for determining the bundle for which each subject votes (steps 7-10). In the remainder of the outermost loop (steps 12-16), the label of each input group is decided. To execute the algorithm on the GPU, the data such as mean vectors, covariance matrices, and inverse covariance matrices, for each input group and example group, are copied to the GPU memory. A single block is assigned to the computation of an SKLD.

Let B be the maximum (allowed) number of blocks in a grid and M the sum of the numbers of groups in all example subjects. Since M blocks are required to compute all SKLDs for an input tract group, the number of input groups N that can be processed

simultaneously with the grid is given as follows:

$$N = \left\lfloor \frac{B}{M} \right\rfloor \quad (1)$$

Thus, as shown in S2 Fig, we use a two-dimensional grid of $N \times M$ blocks on the GPU for SKLD computation. The first dimension is for the input tract groups which are processed simultaneously with the grid, and the second dimension is for the groups in the example data. Therefore, the SKLDs between N input groups and M example groups are simultaneously computed in the grid, and the results are stored in an $N \times M$ array in the GPU memory.

Each block (i, j) calculates the SKLD between an input tract group g_i and an example tract group b_j using the following equation (also see Eq. (14)):

$$d_{SKL}(g_i, b_j) = \frac{1}{2}(trace(S_i^{-1}S_j) + trace(S_j^{-1}S_i) + v^T S_i^{-1} v + v^T S_j^{-1} v - 2k) \quad (2)$$

To compute this equation in parallel, the number of threads in a block is set to the dimensions k of a tract as shown in S2 Fig. Every thread evaluates Eq. (3) which mainly consists of inner products of vectors. Specifically, each thread t in the block starts with performing a series of inner products of vectors in the equation and storing the result in the t-th location of an array mem in the GPU shared memory:

$$mem(t) = S_i^{-1}(t, \cdot)S_j(\cdot, t) + S_j^{-1}(t, \cdot)S_i(\cdot, t) + v(t)[S_i^{-1}(t, \cdot)v + S_j^{-1}(t, \cdot)v] \quad (3)$$

where $S(t, \cdot)$ and $S(\cdot, t)$ are the t-th row vector and the t-th column vector of a matrix S, respectively, and $v(t)$ is the t-th element of a vector v. As indicated in Eq. (2), the sum of the values in mem is subtracted by $2k$, then divided by two, and finally stored as the SKLD at the (i, j)-th location of an $N \times M$ array in the GPU memory.

Afterwards, the GPU performs the voting scheme by referencing the array that contains the SKLDs computed in the first stage, for determining the labels of N input groups in parallel. Let T be the total number of subjects in the example data. To perform the voting scheme with T example subjects for N input tract groups in parallel, we construct a one-dimensional grid of N blocks, each of which contains T threads, as shown in S3 Fig. For an input tract group g_i, each thread j in block i determines the

most similar tract group in the j-th example subject, by using the precomputed SKLDs between g_i and every tract group in the example subject j, and votes for the bundle containing the most similar tract group. The label of g_i is set to that of the bundle with the majority of votes. The two stages are performed repeatedly until all input tract groups are labeled. The resulting labels for all input tract groups are copied back to the CPU memory to complete input group labeling.
