# Supplementary figures and images for: An Example-Based Multi-Atlas Approach to Automatic Labeling of White Matter Tracts
Source: PLoS One. 2015 Jul 30;10(7):e0133337. doi: 10.1371/journal.pone.0133337 (PMC4520495; doi:10.1371/journal.pone.0133337)

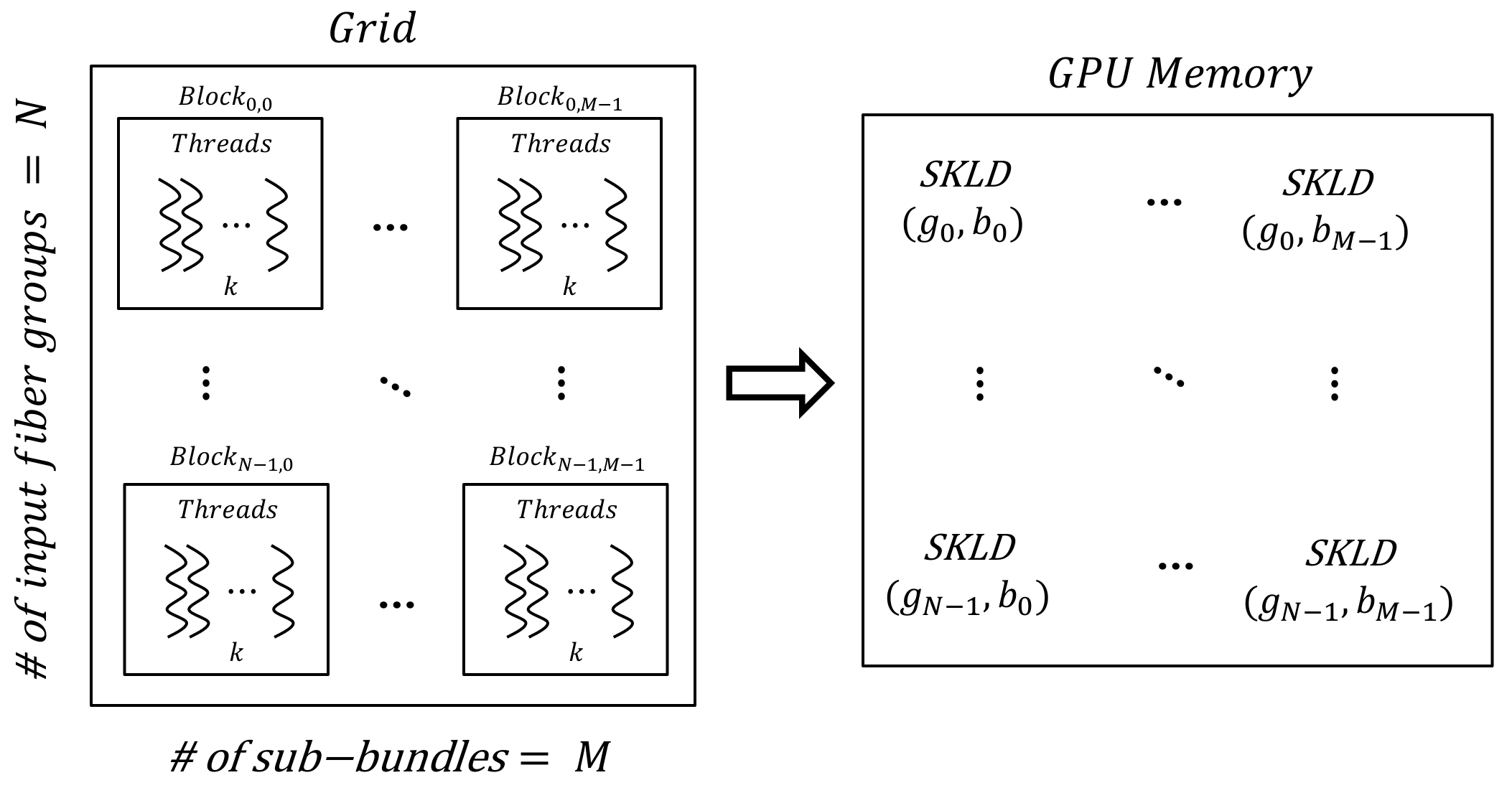

Supplement: S1 Fig — In the first stage, the grid consists of N × M blocks, and each block contains k threads. A block (i, j) is used for computing the SKLD between an input tract group g i and an example tract group b j. The resulting SKLDs are stored in an N × M array in the GPU memory. (TIF) [file pone.0133337.s002.tif]

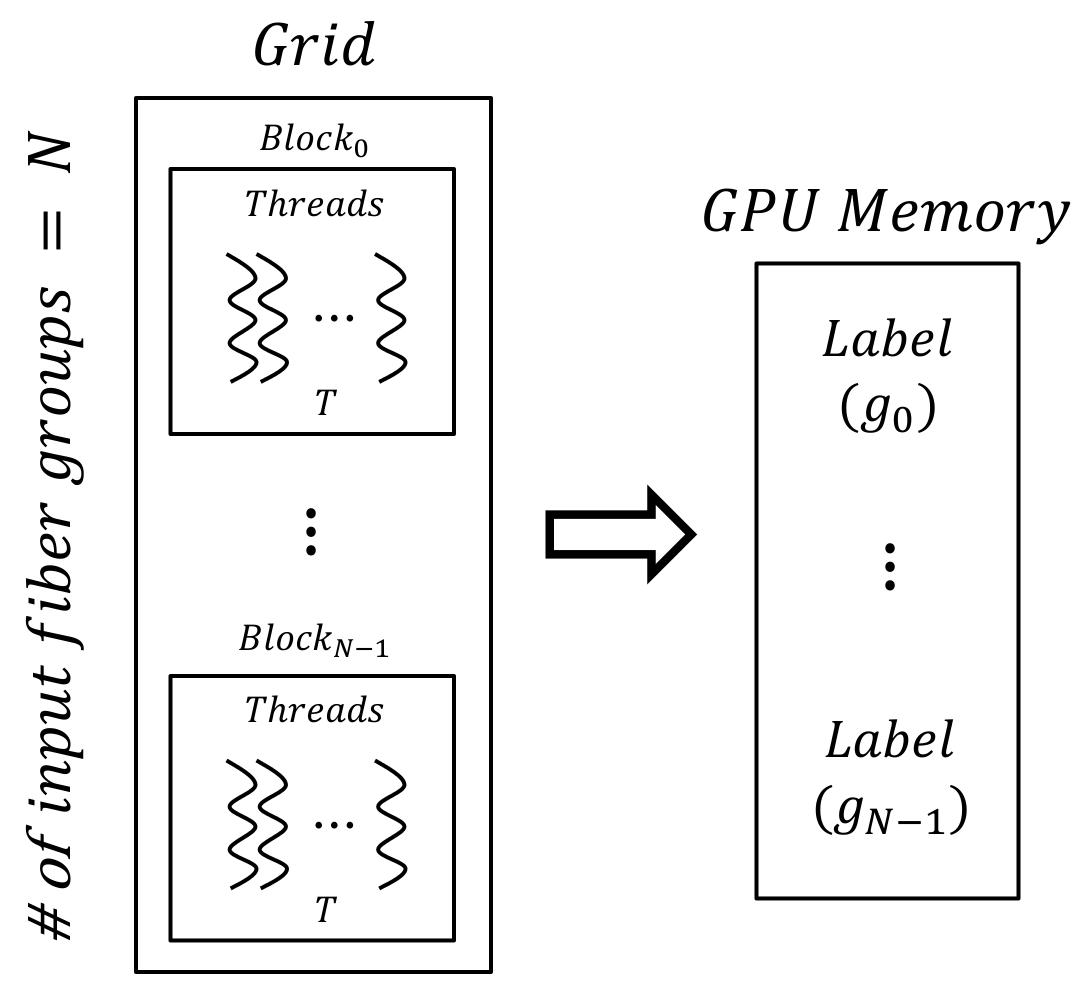

Supplement: S2 Fig — In the second stage, the grid consists of N blocks, and each block contains T threads. The threads in a block are used to label an input group g i based on voting scheme. The resulting labels are stored in the GPU memory space of size N. (TIF) [file pone.0133337.s003.tif]
